# Comparative assessment of deep belief network and hybrid adaptive neuro-fuzzy inference system model based on a meta-heuristic optimization algorithm for precise predictions of the potential evapotranspiration

**DOI:** 10.1007/s11356-024-33987-3

**Published:** 2024-06-15

**Authors:** Muhammed Ernur Akiner, Mehdi Ghasri

**Affiliations:** 1 Vocational School of Technical Sciences, Department of Environmental Protection Technologies, Akdeniz University, Antalya, Turkey; 2 Department of Civil Engineering, University of Sistan and Baluchestan, Zahedan, Iran

**Keywords:** Adaptive network-based fuzzy inference system, Deep belief network (DBN), Forecasting, Hybrid artificial intelligence, Potential evapotranspiration, Snake optimizer

## Abstract

Accurately predicting potential evapotranspiration (PET) is crucial in water resource management, agricultural planning, and climate change studies. This research aims to investigate the performance of two machine learning methods, the adaptive network-based fuzzy inference system (ANFIS) and the deep belief network (DBN), in forecasting PET, as well as to explore the potential of hybridizing the ANFIS approach with the Snake Optimizer (ANFIS-SO) algorithm. The study utilized a comprehensive dataset spanning the period from 1983 to 2020. The ANFIS, ANFIS-SO, and DBN models were developed, and their performances were evaluated using statistical metrics, including *R*^2^, ${R}_{adj}^2$, NSE, WI, STD, and RMSE. The results showcase the exceptional performance of the DBN model, which achieved *R*^2^ and ${R}_{adj}^2$ values of 0.99 and NSE and WI scores of 0.99 across the nine stations analyzed. In contrast, the standard ANFIS method exhibited relatively weaker performance, with *R*^2^ and ${R}_{adj}^2$ values ranging from 0.52 to 0.88. However, the ANFIS-SO approach demonstrated a substantial improvement, with *R*^2^ and ${R}_{adj}^2$ values ranging from 0.94 to 0.99, suggesting the value of optimization techniques in enhancing the model’s capabilities. The Taylor diagram and violin plots with box plots further corroborated the comparative analysis, highlighting the DBN model’s superior ability to closely match the observed standard deviation and correlation and its consistent and low-variance predictions. The ANFIS-SO method also exhibited enhanced performance in these visual representations, with an RMSE of 0.86, compared to 0.95 for the standard ANFIS. The insights gained from this study can inform the selection of the most appropriate modeling technique, whether it be the high-precision DBN, the flexible ANFIS, or the optimized ANFIS-SO approach, based on the specific requirements and constraints of the application.

## Introduction

Turkey’s Antalya region is well-known for its varied climate and distinctive weather patterns. The area is divided into many districts, each with unique meteorological insights: Antalya Centrum, Manavgat, Alanya, Gazipaşa, Korkuteli, Elmalı, Finike, Demre, and Kaş. Antalya’s and its districts’ meteorological insights offer many study opportunities, including investigations into the consequences of climate change, vegetation, genetic variety, greenhouse heating needs, and local climate vulnerabilities. Comprehending the complex interplay among climate, topography, and indigenous ecosystems is vital for proficient environmental governance and sustainable growth in the area.

Research on the amount of water lost by evapotranspiration in various ecosystems sheds light on these processes’ dynamics. For example, Özhan et al. (2010) study on evapotranspiration from Turkish forest ecosystems describes the techniques used to calculate evapotranspiration and compares it with potential evapotranspiration. Furthermore, studies have been conducted in many locations, including southeast Turkey, on variations in potential evapotranspiration brought on by meteorological conditions (Özdoğan and Salvucci 2004). These studies highlight the correlation between potential evapotranspiration and environmental parameters such as humidity and wind speed. Moreover, Muratoglu’s (2020) evaluation of Turkey’s virtual water trade and wheat’s water footprint highlights the regional variations in crop evapotranspiration estimates. The province of Antalya and its districts’ potential for evaporation and evapotranspiration is impacted by many environmental factors, such as irrigation techniques, land use, and climate change. To maintain sustainable management of water resources in the face of changing environmental conditions, stakeholders can make informed decisions by synthesizing research findings on evapotranspiration dynamics in various ecosystems and locations.

Evapotranspiration potential is a critical factor that significantly influences the region’s agricultural output and water resource management. Evapotranspiration is essential in establishing the total hydrological dynamics and the amount of water crops require. It is crucial to comprehend Antalya’s and its districts’ evapotranspiration patterns to optimize irrigation techniques, distribute water resources, and manage land use sustainably. By thoroughly studying the meteorological data pertaining to evapotranspiration potential in Antalya province and its districts, this research seeks to illuminate the complex interplay between agricultural practices and climate dynamics. Different climatic features are found in Antalya, a province in Turkey, and its districts, including Antalya Centrum, Manavgat, Alanya, Gazipaşa, Korkuteli, Elmalı, Finike, Demre, and Kaş. Research by Güney et al. (2019) has indicated that Antalya has frigid winters and dry summers due to its oro-Mediterranean climate. Research on the impact of climate change on the region’s severe rainfalls has shown that these events are non-stationarous (Yilmaz 2015; Yilmaz 2017). Understanding local climate vulnerabilities is crucial because of the influence of climate change on a variety of factors, including agricultural pests (Kutywayo et al. 2013), erosion and land cover changes (Coşgun 2017), and health consequences (Mahapatra et al. 2022). These effects have been examined in numerous districts worldwide.

Comprehending how climate change affects evapotranspiration and water demand is essential to managing water resources sustainably. Research on how crop growth and irrigation demand are affected by climate change in Mediterranean regions, such as Turkey (Yano et al. 2007), offers important insights into future changes in evapotranspiration. Furthermore, the research by Gelati et al. (2020) on the sustainability of groundwater irrigation in the Euro-Mediterranean region emphasizes the anticipated changes in potential evapotranspiration and precipitation, highlighting the necessity of adaptive water management techniques. The possible effects of drought on vegetation make areas like Antalya, Turkey, and its districts extremely concerned. According to Messinger et al. (2015), Cedrus libani, also called Cedar of Lebanon, has demonstrated the potential to withstand harsh environmental circumstances such as intense winter cold and protracted summer droughts. This drought tolerance is essential in places like Antalya and its districts, where water shortage can limit plant growth and survival. Studies on warm-season turfgrasses’ ability to withstand dryness in Turkey’s Mediterranean area, which includes Antalya, emphasize how crucial soil moisture content is to plant survival and growth (Severmutlu et al. 2011). Furthermore, research on how soil features affect water retention is highlighted by studies on how soil type and compaction conditions affect soil water characteristics (Miller et al. 2002).

Crop output in Turkey has been determined to benefit from implementing agricultural assistance programs throughout the long and short term (Canbay 2021). Compared to many other regions, the region’s agricultural production is notable due to its high crop variability and yields (Satir and Yeler 2016). As stated by Erinç and Tunçdilek (1952), the highly fertile soil of Antalya is used to cultivate a wide variety of goods, including wheat, corn, sesame, cotton, oranges, and bananas. Cut flowers are also grown and exported. Most coastal regions are used to cultivate cotton, peanuts, sesame, citrus fruits, bananas, and early-season vegetables. Grains, legumes, sugar beets, grapes, quinces, and pears are among the fruits grown in the region’s interior. The production of greenhouses in the Antalya Region has grown significantly in recent years. According to Gözener and Dereli (2018), 61.72% of Antalya’s greenhouse output is produced by tomatoes, which dominate the region’s agricultural landscape. As a significant contributor to the local economy, the area is well-known for its Besni pepper (Capsicum annuum L.) (Şahin et al. 2022).

Turkey, especially the Antalya region, has mostly thermic or mesic soil types (Tekkanat and Öztürk 2022). Due to its ideal soil and climate, Turkey’s East Mediterranean region—where several of these districts are located—is emphasized as a good place for agricultural production (Bozdoğan 2020). However, due to elements like steep slopes, hilly terrain, and improper land use practices, there are worries regarding soil erosion in Turkey, particularly in locations like Elmalı (Aydın and Tecimen 2010).

According to the above, forecasting PET modeling is crucial in understanding the environmental dynamics and sustainable development in Turkey’s Antalya region and its districts. Modeling using artificial neural networks and machine learning algorithms has gained popularity in recent years because they can analyze complex datasets and make accurate predictions based on patterns and relationships within the data (Dou and Yang 2018). Modeling PET using artificial neural networks and machine learning techniques offers significant advantages over traditional methods (analytical models) in understanding and forecasting environmental dynamics, in which researchers can more effectively capture non-linear relationships and intricate interactions among various environmental factors than traditional analytical models. These advanced techniques can handle large datasets with high dimensionality, allowing for a more comprehensive analysis of the factors influencing evapotranspiration in the region (Chen et al. 2020).

One of the key advantages of using artificial neural networks and machine learning for PET modeling is their ability to adapt and learn from new data, continuously improving the accuracy of predictions over time. These models can detect subtle patterns in the data that may not be apparent through traditional analytical approaches, leading to more precise forecasts of potential evapotranspiration in Antalya and its districts. Furthermore, artificial neural networks and machine learning algorithms can handle complex and dynamic environmental systems, making them well-suited for capturing the variability and uncertainties associated with climate change impacts on evapotranspiration patterns. These models can provide insights into how changing environmental conditions, such as temperature variations and precipitation trends, affect evapotranspiration dynamics in the region. In addition to their flexibility and adaptability, artificial neural networks and machine learning models offer scalability, allowing researchers to easily incorporate new data sources and variables into the modeling process. This scalability enables a more holistic understanding of the factors influencing evapotranspiration in Antalya and its districts, leading to more robust decision-making for sustainable water resource management and agricultural practices. By embracing these advanced modeling techniques, researchers can unlock valuable insights into evapotranspiration patterns and contribute to more effective water resource management strategies in the face of changing environmental conditions (Liu et al. 2022).

Integrating optimization algorithms with artificial neural networks and machine learning methods can enhance their efficiency and power in PET modeling. Optimization algorithms play a crucial role in fine-tuning the parameters of neural networks and machine learning models to improve their performance and predictive accuracy. Optimization algorithms can optimize the weights, biases, and hyperparameters of neural networks and machine learning models for PET modeling. These algorithms enable researchers to search for the best parameters that minimize prediction errors and maximize the accuracy of evapotranspiration forecasts. By iteratively adjusting model parameters based on optimization results, researchers can refine their models and achieve better predictions of potential evapotranspiration in the region. Moreover, combining optimization algorithms with artificial neural networks and machine learning methods allows for the development of more robust and adaptive models that can more effectively capture the complexities of environmental systems. These enhanced models can better handle uncertainties, non-linear relationships, and dynamic interactions among environmental factors, leading to more accurate and reliable forecasts of evapotranspiration dynamics (Mostafa et al. 2023).

Therefore, this study attempts to employ both the adaptive network-based fuzzy inference system (ANFIS) and the deep belief network (DBN) machine learning methods and further hybridize the ANFIS approach with the Snake Optimizer (SO) (Hashim and Hussien 2022) optimization algorithm. The primary objectives of this study can be summarized as follows:

- Utilizing a hybrid artificial intelligence (AI) model to predict potential evapotranspiration (PET) as a machine learning task.
- Evaluation of the performance of the developed models, both ANFIS and DBN and the optimized ANFIS-SO method, using comprehensive statistical metrics.

Including the DBN deep learning method alongside the ANFIS approach expands the scope of the study, allowing for a comparative assessment of the strengths and limitations of these two distinct modeling techniques. By incorporating the DBN model, the authors aim to leverage the deep learning paradigm’s ability to capture complex non-linear relationships and potentially outperform the standard ANFIS method in predicting the PET variable.

Furthermore, hybridizing the ANFIS method with the SO optimization algorithm enhances the model’s performance and addresses shortcomings in the standard ANFIS approach. This optimization process may unlock additional predictive capabilities, potentially bridging the gap between the ANFIS and DBN models’ performances and providing a more robust and accurate solution for PET estimation.

The rest of the paper is organized as follows: “Materials and methods” section describes the research area and the instrumentation. The “Methodology” section presents the recommended methodology. The “Results and discussion” section presents the novel methodology’s outcomes and discusses them. The “Conclusions” section summarizes the work and provides recommendations for future research directions.

## Materials and methods

### Study sites and instrumentation

**Fig. 1:**
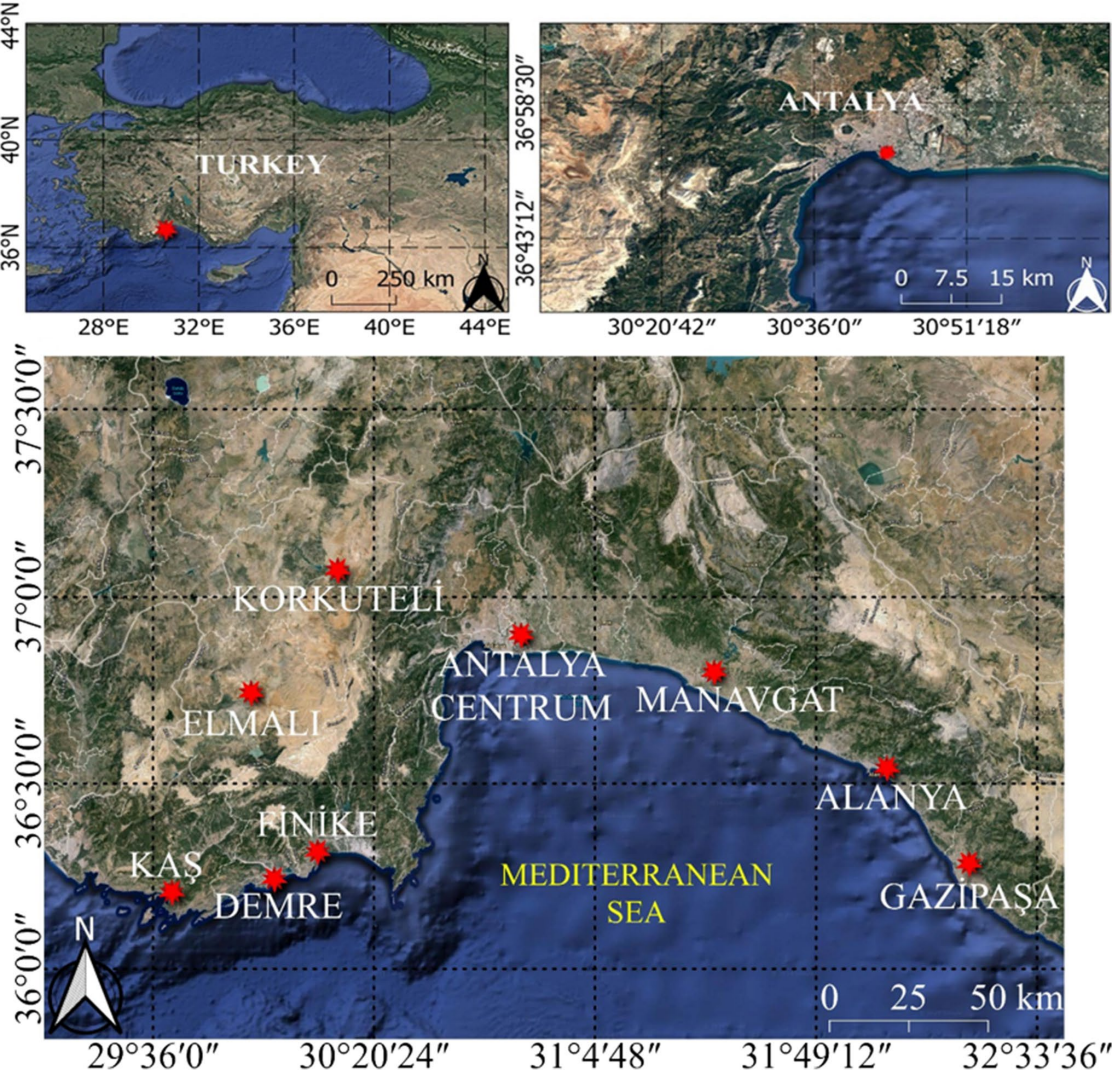
Geographical positions of the nine districts of Antalya

The study area is the Mediterranean coast of Turkey’s Antalya province. Nine districts of Antalya, namely Antalya Centrum, Manavgat, Alanya, Gazipaşa, Korkuteli, Elmalı, Finike, Demre, and Kaş, were studied using meteorological data spanning an extended period. Figure 1 shows the geographical positions of the nine districts of Antalya, and Table 1 shows the precise coordinates of the meteorological stations. Turkey’s General Directorate of Meteorology (MGM) provided the utilized dataset. Meteorological variables and their associated sensors are listed in Table 2.

**Table 1:** Information about meteorological stations

Name	No	Altitude (m)	Observation years	Latitude	Longitude
Antalya Centrum	17300	54	1983–2020	36°54′22.7′′N	30°47′56.4′′E
Alanya	17310	7	1983–2020	36°33′02.7′′N	31°58′49.3′′E
Finike	17375	2	1983–2020	36°18′08.6′′N	30°08′44.9′′E
Kaş	17380	5	1983–2020	36°12′00.6′′N	29°39′00.6′′E
Korkuteli	17926	1014	1983–2020	37°03′23.4′′N	30°11′27.7′′E
Elmalı	17952	1095	1983–2020	36°44′14.1′′N	29°54′43.6′′E
Manavgat	17954	38	1983–2020	36°47′22.3′′N	31°26′27.8′′E
Demre	17970	25	1983–2020	36°14′31.4′′N	29°58′44.4′′E
Gazipaşa	17974	21	1983–2020	36°16′28.9′′N	32°18′27.2′′E

**Table 2:** Meteorological variables and their associated sensors

Solar hour	The Campbell-Stokes heliographs were used.
Solar radiation	A silicon pyranometer was used.
Soil heat flux	A precise infrared temperature sensor monitors the ground surface.
Relative humidity	A capacitive thin-film polymer humidity sensor offers precise and reliable measurements even in highly humid conditions.
Wind speed	A three-cup anemometer and an air temperature shield inlet are situated at the same height.
Air temperature	Three platinum resistance thermometers are contained inside fan-aspirated solar radiation shields.
Rain	A wind-shielded, inlet-heated rain gauge with three load cell sensors, a precipitation or wetness detector, and an additional tipping bucket rain gauge were used.

### Database

**Fig. 2:**
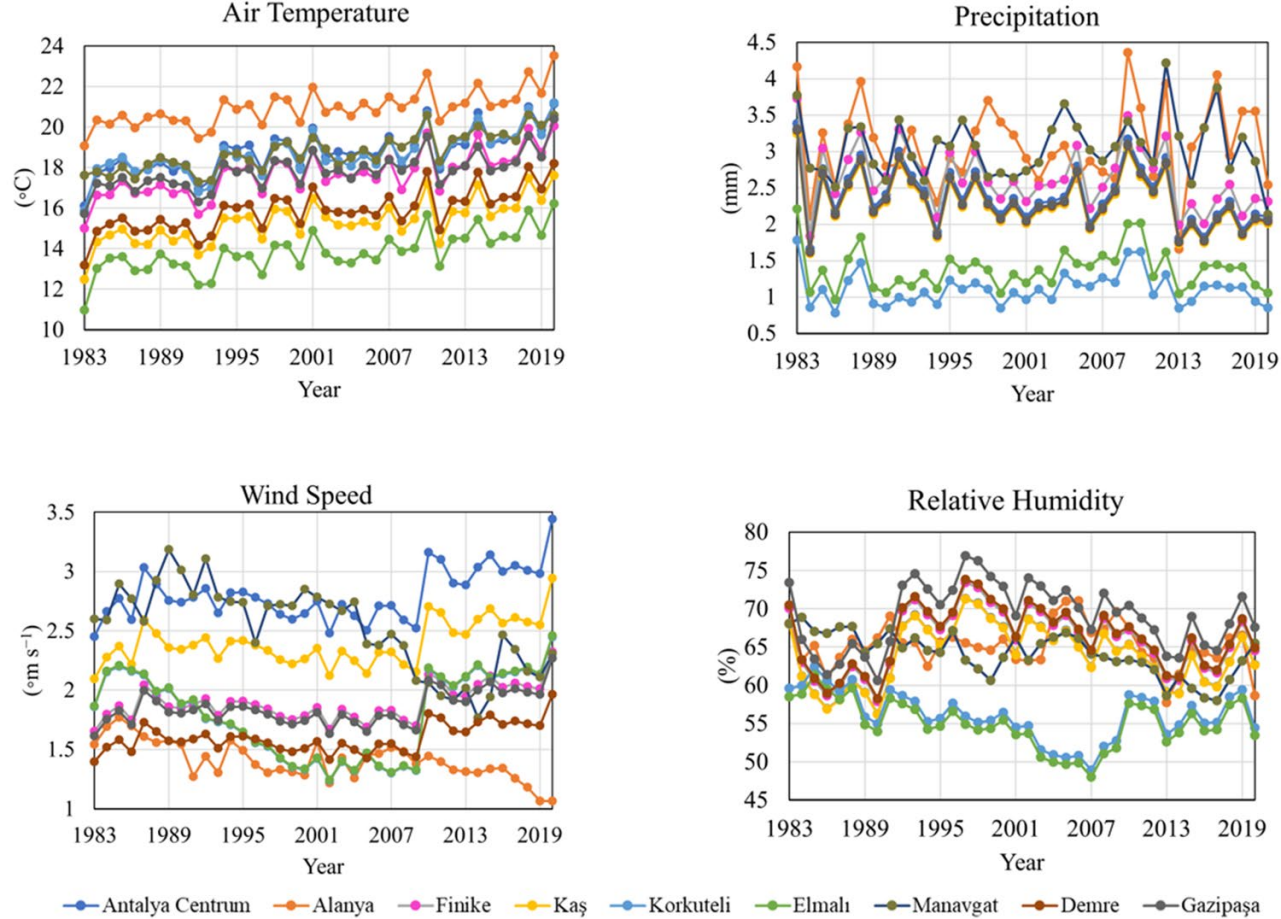
The monthly mean measurements of air temperature, relative humidity, wind speed, and precipitation across the monitoring stations located in Antalya Centrum, Alanya, Finike, Kaş, Korkuteli, Elmalı, Manavgat, Demre, and Gazipaşa

The current database encompasses data gathered from nine stations, namely Antalya Centrum, Alanya, Finike, Kaş, Korkuteli, Elmalı, Manavgat, Demre, and Gazipaşa, from 1983 to 2020. Figure 2 illustrates the average monthly values for temperature, relative humidity, wind speed, and precipitation recorded at these nine locations within this timeframe. Analytical measurements utilized daily computed values from 1983 to 2020, encompassing 135,455 data points. This comprehensive dataset is valuable for analyzing and understanding climatic patterns and trends in the specified regions.

## Methodology

### Potential evapotranspiration methods

The methodology involved in estimating potential evapotranspiration (PET) encompassed the application of various empirical methods, which can be classified into four distinct groups based on the variables they rely on for computation: mass-transfer-based methods, temperature-based methods, radiation-based methods, and combination methods (Xu and Singh 2002). Table 3 provides a compilation of formulas representing these methodological approaches.

**Table 3:** PET methods

Methods	PET equation	Equation	Ref.
Mass-transfer	$\textrm{PET}=0.00536\sqrt{u}\left({e}_s-{e}_a\right)$	(1)	Trabert (1896)
Temperature	$\textrm{PET}=0.736{\left({e}_s-{e}_a\right)}^{0.7}{\left(1+\frac{T_a}{273}\right)}^{4.8}$	(2)	Antal (1968)
Radiation	$\textrm{PET}=a\frac{\Delta }{\Delta +\gamma}\frac{R_n-G}{\lambda}\kern1.75em a=1.82,2.14$	(3)	Tabari and Talaee (2011)
Combination (FAO)	$\textrm{PET}=\frac{0.408\Delta \left({R}_n-G\right)+\gamma \frac{900}{T_a+273}u\left({e}_s-{e}_a\right)}{\Delta +\gamma \left(1+0.34u\right)}$	(4)	Allen et al. (1998)

*Where u is the wind speed at 2 m height in $\frac{m}{s}$, *T*_*a*_ is the daily mean temperature in ^°^C. *e*_*s*_ and *e*_*a*_ are the saturation and actual vapor pressures, respectively, in kPa, except Eq.2, where they are in hPa. *R*_*n*_ is the net radiation fluxes in MJ m^−2^day^−1^, G is the soil heat flux in MJ m^−2^day^−1^, ∆ is the slope of the vapor pressure curve (kPa °C^−1^), γ is the psychrometric constant (kPa °C^−1^), and *λ* = 2.501 – 0.002361 × *T*_*a*_

#### FAO-56 Penman-Monteith

**Fig. 3:**
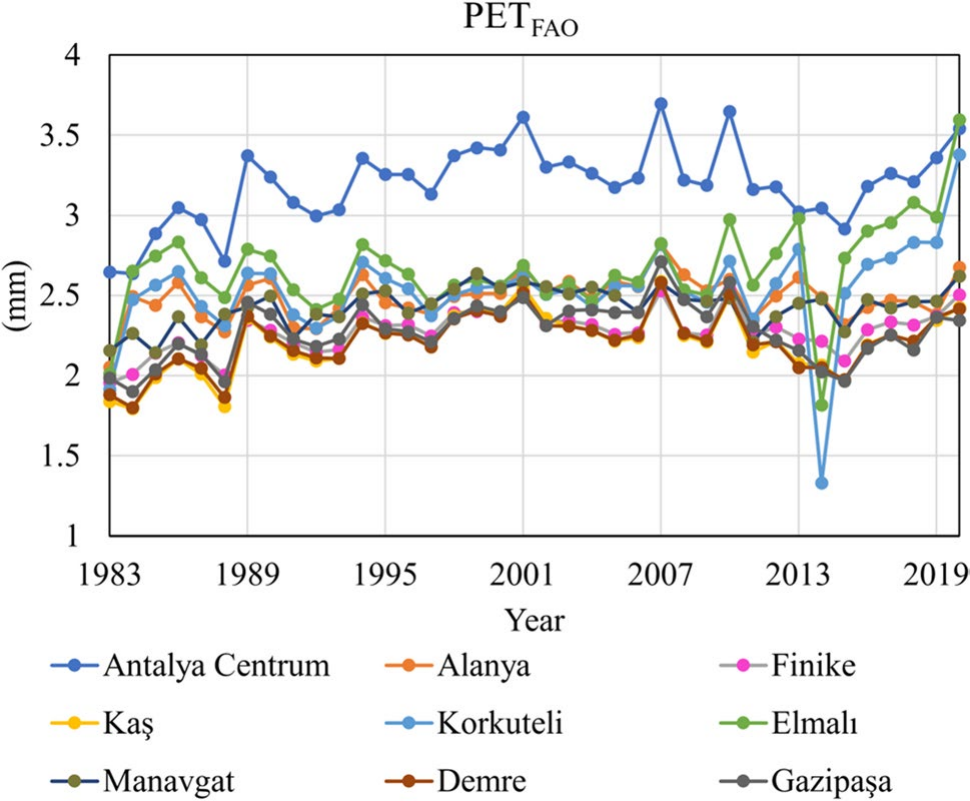
The mean PET value calculated using the FAO method

The FAO-56 Penman-Monteith model, recommended by the Food and Agriculture Organization of the United Nations (FAO) as the standard method for estimating potential evapotranspiration (PET), is widely acknowledged for its applicability across diverse climatic conditions and regions. FAO-PM model integrates energy balance and aerodynamic processes, incorporating aerodynamic and surface resistances to provide accurate PET estimations (Proutsos et al. 2023; Makwana et al. 2023; Yong et al. 2023). The FAO-PM model is founded on physical principles, ensuring precise results through rational relationships. Compared to other empirical models, such as temperature-based, radiation-based, and mass-transfer-based, the FAO-PM method demonstrates superior performance due to its comprehensive approach considering various factors affecting PET calculation (Gentilucci et al. 2021; Chen et al. 2020; Dou and Yang 2018; Liu et al. 2022). The detailed formulae and data requirements for these models are outlined in Table 3, facilitating a thorough understanding of their methodologies and applications in PET estimation. Figure 3 illustrates the mean PET value calculated using the FAO method.

### Machine learning

#### Adaptive network-based fuzzy inference system

The adaptive network-based fuzzy inference system (ANFIS) is a computational model that combines the strengths of fuzzy logic and neural networks to facilitate intelligent decision-making and pattern recognition. ANFIS employs a hybrid learning algorithm that adapts and refines its fuzzy rules and membership functions based on input-output data pairs. This adaptive mechanism allows ANFIS to effectively model complex, non-linear relationships between variables, making it particularly suitable for uncertain and imprecision tasks. ANFIS offers several advantages over conventional artificial neural network (ANN) methods. One key benefit is its ability to interpret and incorporate human-like reasoning through fuzzy logic, enabling a more intuitive and transparent modeling process. Additionally, ANFIS can efficiently handle numerical and linguistic data, enhancing its versatility in various applications compared to traditional ANNs. The adaptive nature of ANFIS allows it to continuously optimize its parameters, leading to improved accuracy and generalization performance, especially in scenarios with limited training data (Chuensiri et al. 2024; Vargas et al. 2024; Jazayeriy and Kazemitabar 2024).

**Fig. 4:**
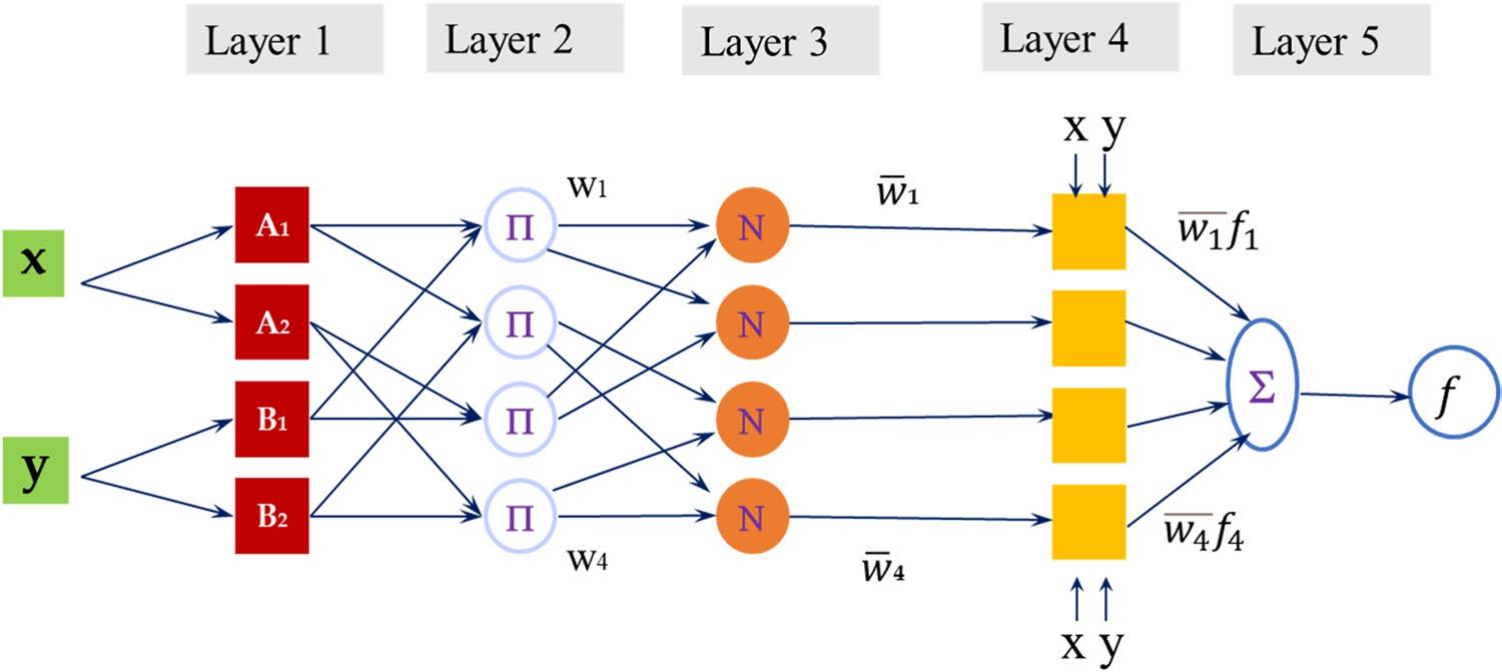
ANFIS structure

In ANFIS, weights and biases play crucial roles in the model’s decision-making process. The weights determine the strength of connections between nodes in the network, influencing the impact of input variables on the system’s output. By adjusting these weights during the learning phase, ANFIS can adapt its structure to effectively capture the underlying patterns in the data. On the other hand, biases introduce flexibility into the model by allowing for shifts in the activation functions of neurons, enabling ANFIS to account for variations and biases in the input data. The dynamic adjustment of weights and biases in ANFIS allows the system to learn and adapt to complex relationships, accurately enhancing its performance in modeling real-world phenomena (Ghasemi et al. 2024; Singh et al. 2012). According to Fig. 4, the concept of ANFIS structure consists of five distinct layers (Singh et al. 2009; Catalão et al. 2010; Yildirim et al. 2023).

##### Layer 1—Fuzzification

The model classifies the inputs based on fuzzy membership functions (MFs) with adjustable parameters in this layer. Because the aim is to reduce the network error using gradian-based methods, the MFs must be differentiable in this layer. These functions can usually be triangular, trapezoidal, Gaussian, or bell-shaped. The membership functions considered in this study are Gaussian.

##### Layer 2—Rules

The rule operator (AND/OR) is applied so that an output represents the previous results for a fuzzy rule that multiplies the received signals. In short, this layer defines the weight of MFs.

5 $${w}_i={\mu}_{Ai}(x)\times {\mu}_{Bi}(y),i=1,2$$

Where:

[*w*_*i*_]→the firing strength of the rule.

Each output node of this layer represents the level of correctness or the degree of truth of the law.

##### Layer 3—Normalization

In this layer, the correct level of the rules is normalized. This layer represents the strength of the i-th rule to the sum of the strength of all the rules.

6 $$\overline{w}=\frac{w_i}{w_1+{w}_2},i=1,2$$

Where:

$\left[\overline{w}\right]$→the normalized firing strength of the rule.

##### Layer 4—Defuzzification

A layer executes the output, resulting from the inference of rules, and multiplies them with the Sugeno fuzzy rule’s function.

7 $$\overline{w_i}{f}_i={w}_i\left({p}_ix+{q}_iy+{r}_i\right)$$

Where:

$\left[\overline{w_i}\right]$→the output of layer 3.

[*p*_*i*_, *q*_*i*_, *r*_*i*_]→the coefficients of a linear combination in the Sugeno inference system.

The parameters *p*_*i*_, *q*_*i*_, and *r*_*i*_ are called consequent parameters that can be modified. The parameters of this layer and the first layer are optimized during the network training process.

##### Layer 5—Cumulative

That comprises the weighted average summation method to calculate the network output.

8 $$f=\sum_i\overline{w_i}{f}_i=\frac{\sum_i{w}_i{f}_i}{\sum_i{w}_i}\kern0.5em \to \textrm{Estimated}\ \textrm{overall}\ \textrm{output}$$

The inclusion of the ANFIS model is motivated by its proven effectiveness in various environmental modeling and forecasting tasks, showcasing its ability to capture both linguistic and numerical information, which can provide a unique perspective and potentially complement the capabilities of more advanced techniques.

#### Snake Optimizer

The Snake Optimizer (SO) algorithm was introduced by Hashim and Hussien (2022) based on the behavior of snakes in mating and finding food. It starts with an initial population of snakes, divided into males and females. Each iteration finds the best individual candidate solution by analyzing each group for the best male and best female. The exploration phase represents environmental factors, while the exploitation phase includes evolution phases to make the global optimal efficient. The snakes focus only on eating if food is available, but the temperature is high. In the last stage, the snakes mate if there is food and a cold area, leading to the best solution for that repetition. This algorithm finds the optimal solution in less time than other compared algorithms (Klimov et al. 2020).

Building upon the ANFIS method, the hybridization with the SO algorithm represents an attempt to enhance the performance of the standard ANFIS approach. The optimized ANFIS-SO method aims to leverage the strengths of both the ANFIS and the optimization algorithm to tune the internal parameters and structure to improve the model's predictive accuracy and generalization capabilities (Halima et al. 2024).

#### Enhancing machine learning efficiency through parameter optimization

The effectiveness of machine learning techniques hinges on the precise configuration of their parameters. As the number of input parameters increases, the operational complexity escalates, resulting in a broad spectrum of parameter selection. This expansion augments the search space, posing challenges in identifying appropriate parameters. Consequently, optimization strategies are necessary to ascertain optimal values (Yang and Shami 2020). Continuing further into the optimization process, the initialization stage lays the foundation for subsequent adjustments. Random initialization of weights and biases ensures that the network starts with diverse values, enabling a broad exploration of the solution space. This randomness prevents the optimization process from being trapped in local minima and promotes the discovery of more optimal weight configurations (Wu et al. 2019).

**Fig. 5:**
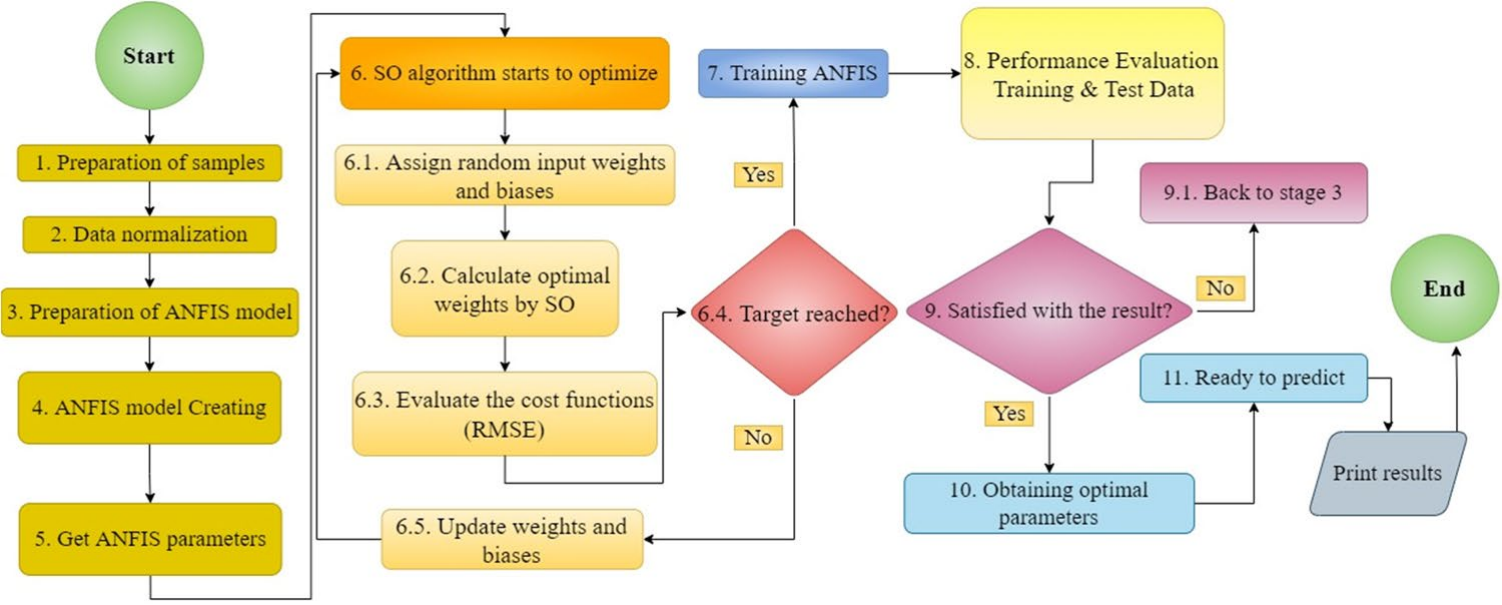
The flowchart of the ANFIS-SO

Once initialized, the next step involves defining a cost function that quantifies the dissimilarity between the network’s predictions and the ground truth values in the training data. The choice of cost function is pivotal, as it guides the optimization algorithm toward minimizing this disparity and improving the model’s predictive accuracy. Here, the RMSE method is used as a cost function. Selecting an appropriate optimization algorithm is a crucial decision in the optimization journey. Different algorithms offer unique approaches to updating weights and biases based on the computed error. The selection of the most suitable algorithm depends on factors like the complexity of the optimization problem, the dataset characteristics, and the computational resources available (Hossain and Timmer 2021). In this context, the meta-heuristic SO algorithm was selected to execute this process, culminating in the development of hybrid methodologies like ANFIS-SO. Setting hyperparameters like learning rate, population size, or convergence criteria fine-tunes the optimization process, ensuring a balance between exploration and exploitation during weight and bias adjustments. These hyperparameters dictate how quickly the network learns from data, how extensively it explores the solution space, and when the optimization process should halt upon reaching a satisfactory solution (Abdul Samad et al. 2023). As the optimization iterations progress, the weights and biases of the ANFIS network undergo continuous refinement based on feedback from the cost function and the optimization algorithm. This iterative process refines the model’s parameters, gradually converging toward a configuration that minimizes prediction errors and maximizes predictive accuracy. The overarching trend of the proposed techniques is depicted in Fig. 5.

### Deep learning

#### Deep belief networks

**Fig. 6:**
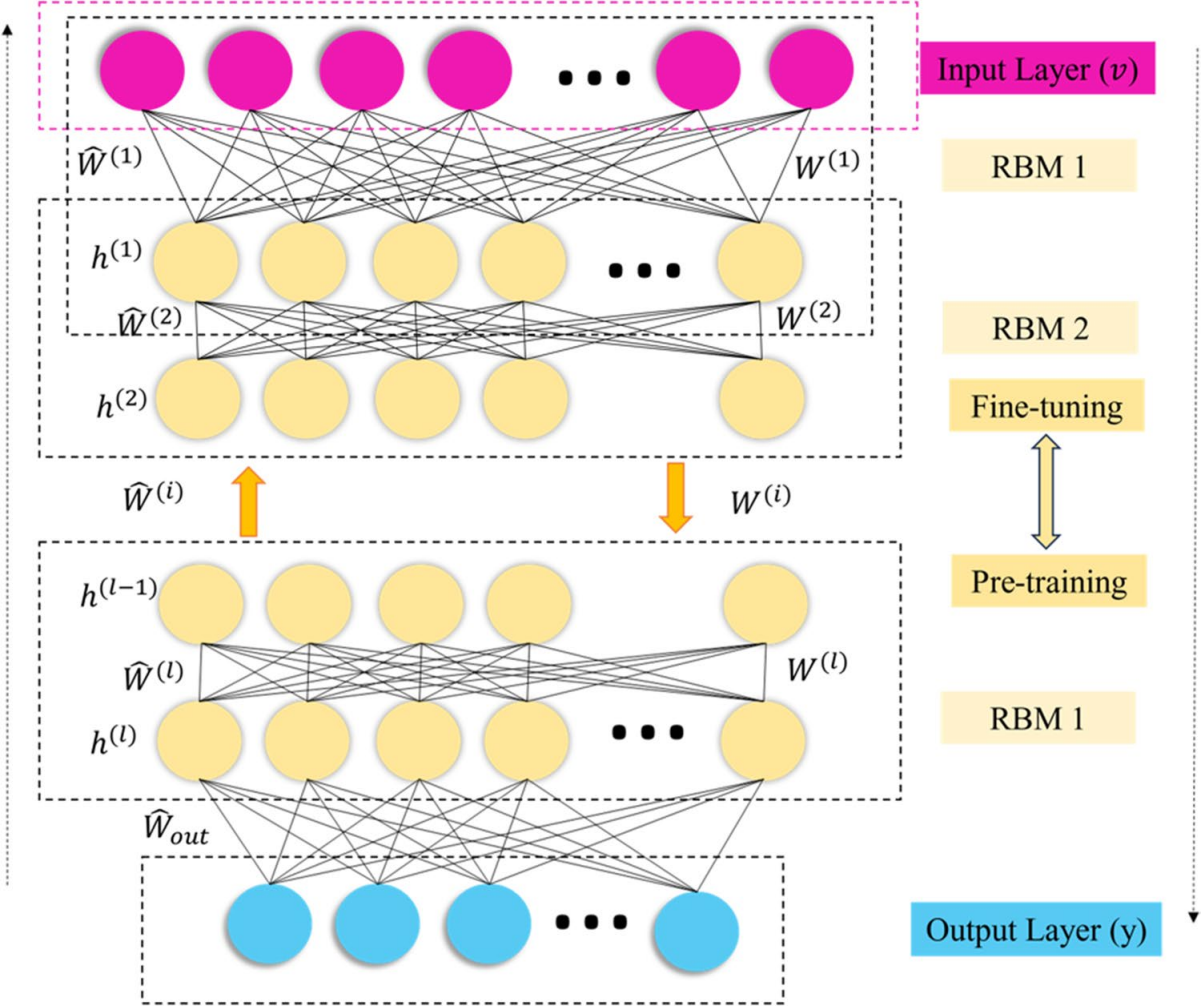
The structural framework of DBN

Deep belief networks (DBNs) (Lopes et al. 2015) are a type of deep neural network that consists of multiple layers of restricted Boltzmann machines (RBMs). RBMs are unsupervised generative models that can learn to extract meaningful features from the input data in an unsupervised manner. The DBN is constructed by stacking multiple RBMs, where the hidden layer of one RBM serves as the visible layer for the next RBM. This hierarchical structure allows the DBN to learn increasingly complex representations of the input data. The training of a DBN is typically done in two stages: (1) Greedy layer-wise pre-training, where each RBM is trained independently in an unsupervised manner, and (2) Fine-tuning, where the entire DBN is trained in a supervised manner to optimize the desired objective function, such as prediction accuracy. Figure 6 exemplifies the structural framework of deep belief networks. This multilayered architecture comprises L hidden layers, with each layer characterized by a weight matrix, *W*^*i*^, that governs the connections between the units of adjacent layers (*i* − 1 and *i*). Additionally, the variables *v* and *h*^*l*^, denote the states of the visible and hidden units within this network topology (Yue et al. 2023; Zhang et al. 2024).

The inclusion of DBN model is motivated by the need to leverage the exceptional performance of deep learning techniques in capturing non-linear relationships and extracting intricate features from data, which are particularly well-suited for modeling complex environmental phenomena. The DBN’s ability to learn hierarchical representations of the input data can potentially uncover hidden patterns and relationships that may not be readily apparent through traditional machine-learning methods, making it a valuable addition to the comparative assessment.

### Models configuration

#### Models initial parameter

The parameter values selected for the ML-based methods to be implemented for predicting potential evapotranspiration are as follows:

The ANFIS model was configured with three membership functions per input variable, and the fuzzy c-means clustering algorithm was utilized with a fuzziness exponent of 2.0, a maximum of 500 epochs, and a minimum improvement threshold of 1e-5. For the ANFIS-SO hybrid approach, the authors employed a population size of 100 and a maximum of 500 iterations for the Snake Optimizer. The authors recommend using a 4-layer architecture for the DBNs, with the number of neurons in each restricted Boltzmann machine (RBM) layer decreasing from 7 to 100 to 50 to 25, and finally to 1 for the output. The learning rate for pre-training the RBMs is set to 0.05, and the learning rate for fine-tuning the DBN is set to 0.005. A batch size of 64 and 500 training epochs were chosen to balance model performance and training time.

#### Data set segmentation

**Fig. 7:**
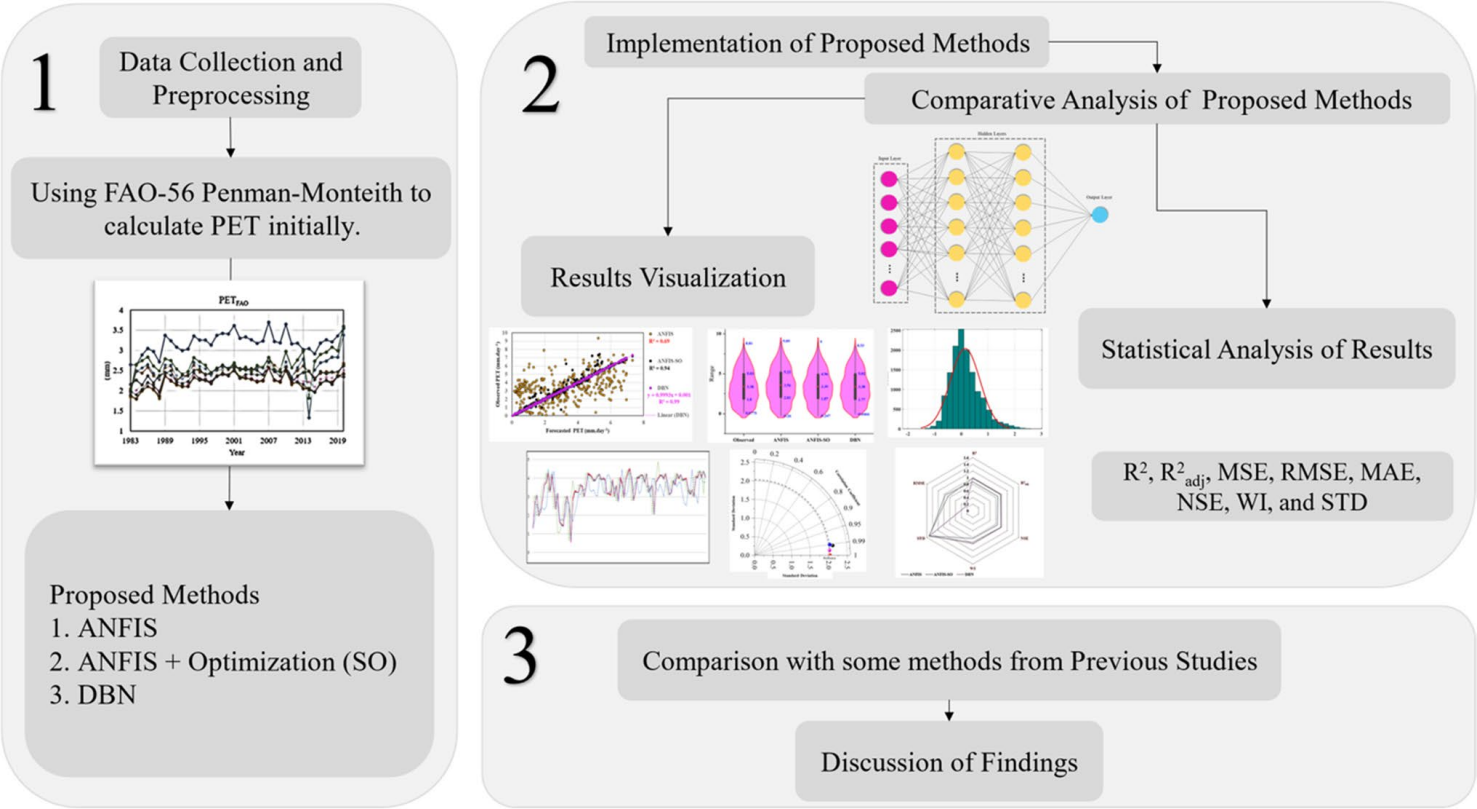
Flowchart of a comparative study on precise prediction of potential evapotranspiration using fuzzy and deep learning methods

The dataset utilized in the study was partitioned into distinct subsets to facilitate the forecasting of PET through the developed modeling approaches. The temporal period from 1983 to 2009 was allocated for the training phase, providing the necessary data to calibrate and optimize the models. The subsequent period, spanning 2010 to 2018, was designated for the testing procedures, enabling the evaluation of the model’s predictive performance on unseen data. Furthermore, covering 2019 to 2020 was reserved as an unseen dataset dedicated to the comparative analysis of the models, particularly the deep learning-based approach (“Performance of deep learning models” section), against this novel set of observations. The partitioning of the dataset into distinct subsets for training, testing, and unseen data provides a comprehensive assessment of the model’s predictive power, its ability to capture the underlying patterns and trends within the data, and its adaptability to novel, previously unseen conditions. The schematic representation of the study’s workflow is presented in Fig. 7.

### Evaluation indicators

This study employed five models to forecast potential evapotranspiration (PET). Various evaluation metrics, including the coefficient of determination (*R*^2^), adjusted coefficient of determination (${R}_{adj}^2$), mean square error (MSE), root mean square error (RMSE), mean absolute error (MAE), Nash-Sutcliffe efficiency (NSE), Wilmot Index (WI), and standard deviation (STD) were utilized to assess the accuracy of the predictive models (Rezaei et al. 2024). The assessment methods are presented in Table 4, where equations 9 to 15 provide the values for *R*^2^, ${R}_{adj}^2$, RMSE, and STD, respectively.

**Table 4:** Details of statistical indices

Methods	Abbreviation	Lower bound	Upper bound
Coefficient of determination	*R*^2^	0	1
Adjusted coefficient of determination	${R}_{adj}^2$	0	1
Root Mean Square Error	RMSE	0	+∞
Mean Absolute Error	MAE	0	+∞
Nash-Sutcliffe efficiency	NSE	−∞	1
Wilmot Index	WI	0	1
Standard deviation	STD	0	+∞

9 $${R}^2=\frac{{\left[\sum_{i=1}^N\left({PET}_{Theo,i}-{PET}_{Theo, avg}\right)\left({PET}_{Exp,i}-{PET}_{Exp, avg}\right)\right]}^2}{\sum_{i=1}^N{\left({PET}_{Theo,i}-{PET}_{Theo, avg}\right)}^2\sum_{i=1}^N{\left({PET}_{Exp,i}-{PET}_{Exp, avg}\right)}^2}$$

10 $${R}_{adj}^2=1-\frac{\left(1-{R}^2\right)-\left(N-1\right)}{N-J-1}$$

11 $$RMSE=\sqrt{\frac{1}{N}\sum_{i=1}^N{\left({PET}_{Theo,i}-{PET}_{Exp,i}\right)}^2}$$

12 $$MAE=\frac{1}{N}\sum_{i=1}^N\left|{PET}_{Theo,i}-{PET}_{Exp,i}\right|$$

13 $$NSE=1-\left[\frac{\sum_{i=1}^N{\left({PET}_{Theo,i}-{PET}_{Exp,i}\right)}^2}{\sum_{i=1}^N{\left({PET}_{Theo,i}-{PET}_{Theo, avg}\right)}^2}\right]$$

14 $$WI=1-\left[\frac{\sum_{i=1}^N{\left({PET}_{Theo,i}-{PET}_{Exp,i}\right)}^2}{\sum_{i=1}^N{\left(\left|{PET}_{Theo,i}-{PET}_{Theo, avg}\right|+\left|{PET}_{Exp,i}-{PET}_{Exp, avg}\right|\right)}^2}\right]$$

15 $$STD=\sqrt{\frac{\sum_1^N{\left(\frac{\ {PET}_{Theo,i}}{PET_{Exp,i}}-\frac{PET_{Theo, avg}}{PET_{Exp, avg}}\right)}^2}{N-1}}$$

Where *N* represents the number of data points, *Theo* signifies the predicted values, *Exp* denotes the experimental (observed) values, *avg* represents the average of the data, and *J* illustrates the number of predictor parameters.

### Normalization of dataset

**Fig. 8:**
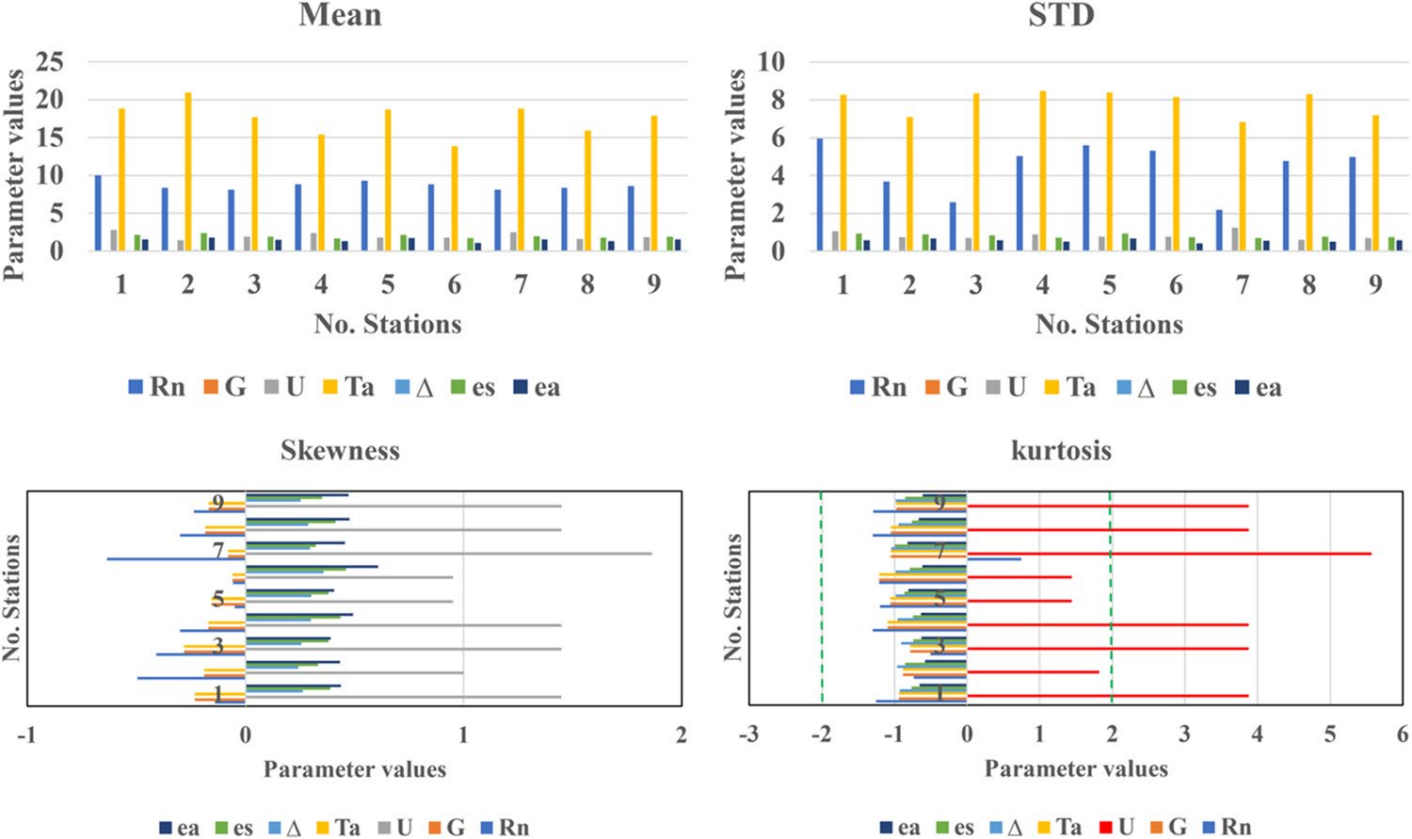
The dataset’s mean, STD, skewness, and kurtosis

The mean, standard deviation (STD), skewness, and kurtosis of the dataset were computed and are presented in Fig. 8. The stations in Antalya Centrum, Alanya, Finike, Kaş, Korkuteli, Elmalı, Manavgat, Demre, and Gazipaşa have been assigned numerical designations ranging from 1 to 9 to enhance their representation in Fig. 8. Normalization is recommended when the skewness and kurtosis of a dataset fall outside the range of − 2 to 2 (Ghasemzadeh Mahani et al. 2021). In the case of the dataset under consideration, normalization is necessary, as indicated in Fig. 6. The Min-Max normalization technique is employed for this purpose (Ghasemzadeh Mahani et al. 2021). Normalization of a dataset involves scaling the values to a standard range, typically between 0 and 1, to ensure uniformity and comparability among different variables or datasets. Min-max normalization scales explicitly the data to a specified range, often between 0 and 1, by subtracting the minimum value and dividing by the range of values. This process aids in mitigating the effects of varying scales and facilitates meaningful comparisons and analyses across datasets. The input parameters for the models are as follows: *U* represents the wind speed at a height of 2 m. *T*_*a*_ denotes the daily mean temperature. *e*_*s*_ and *e*_*a*_ represent the saturation vapor pressure and the actual vapor pressure, respectively. *R*_*n*_ signifies the net radiation fluxes, and *G* indicates the soil heat flux.

## Results and discussion

Before analyzing the outcomes, it is essential to highlight that lower MSE, RMSE, and MAE values signify reduced errors and optimal model performance. Similarly, NSE and WI values nearing one indicate minimal errors and optimal model performance. A smaller disparity between the observed data and the model’s standard deviation suggests decreased errors and enhanced model performance. Furthermore, as the coefficient of determination (*R*^2^) values approach unity, indicating a stronger correlation between the observed values and the model.

Furthermore, the root mean square error (RMSE), mean absolute error (MAE), and standard deviation (STD) share the same unit as the output variable, which is millimeters per day (mm day^-1^). The coefficient of determination (*R*^2^) and adjusted coefficient of determination (${R}_{adj}^2$) are dimensionless quantities, as they represent the proportion of variance in the dependent variable that is predictable from the independent variable(s). The Nash-Sutcliffe efficiency (NSE) and Wilmott Index (WI) are dimensionless metrics, ranging from negative infinity to 1 and 0 to 1, respectively, where 1 indicates a perfect fit or match between the observed and predicted values.

### Performance of ANFIS model

**Fig. 9:**
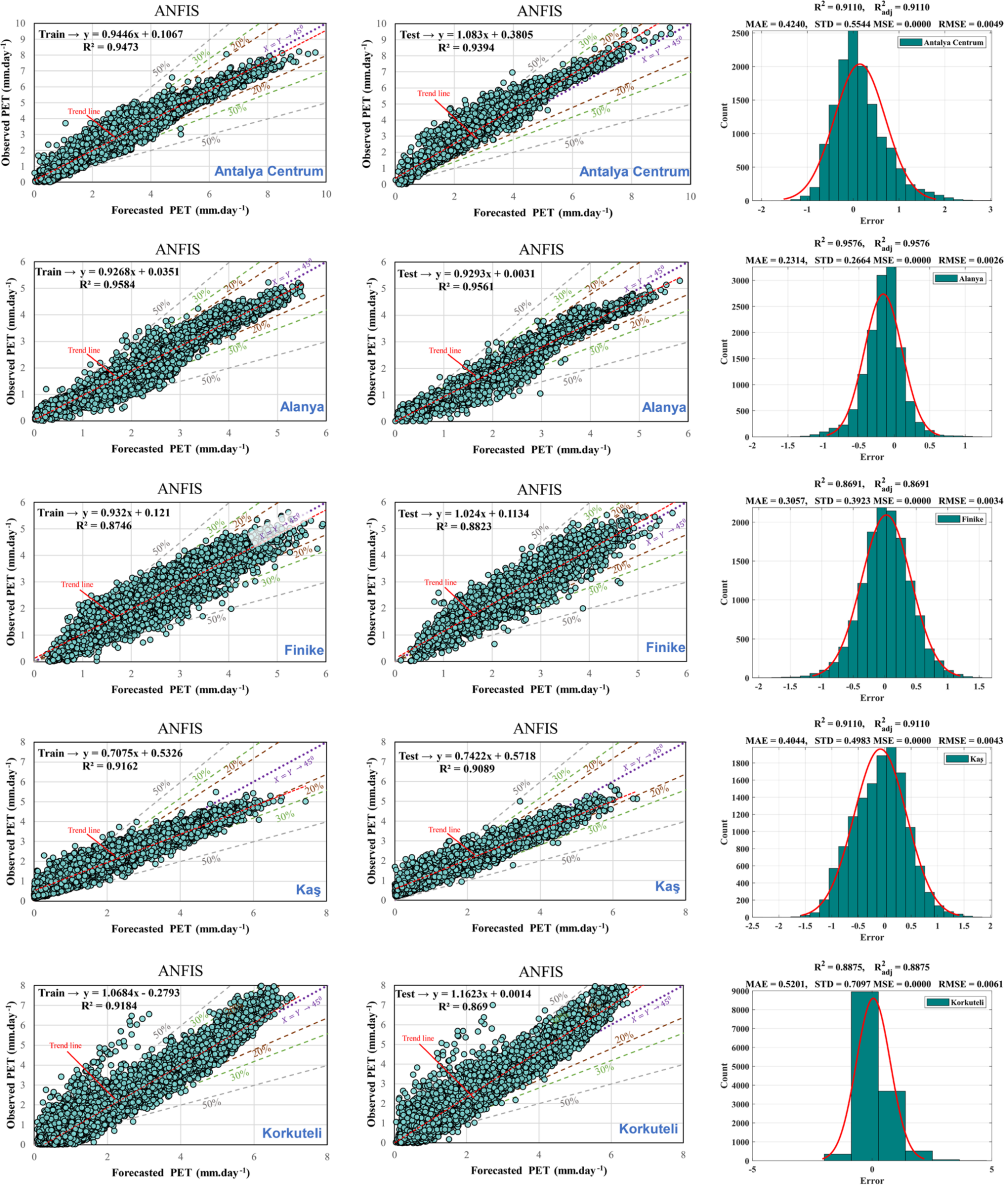

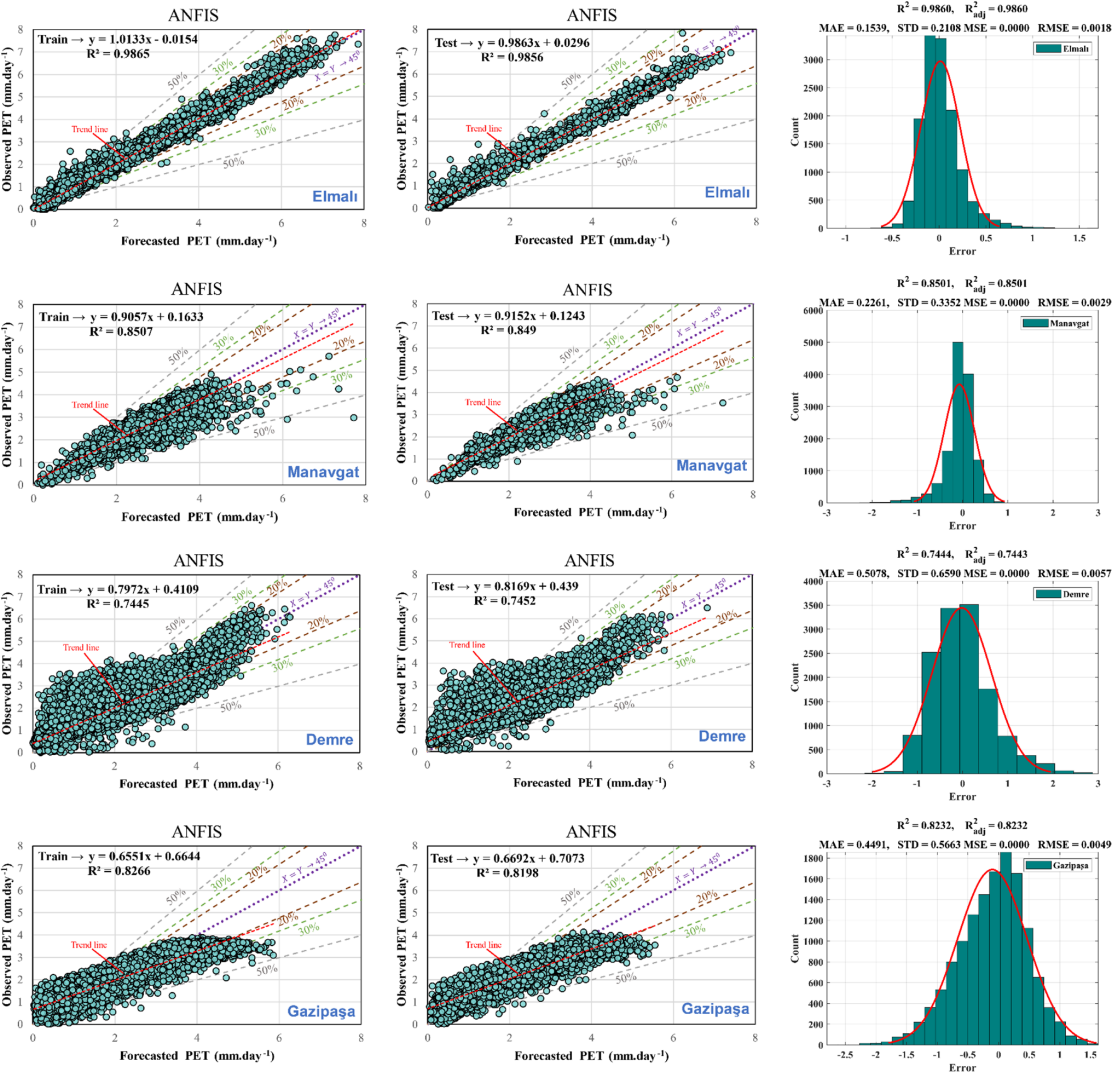
Scatter plot for training and testing phase and histogram errors for all data in ANFIS models

Figure 9 and Table 5 illustrate the effectiveness of the ANFIS model concerning training and testing datasets, presenting outputs, error analysis, scatter plots, and statistical performance. The findings across all nine stations exemplify the ANFIS model’s adeptness in capturing correlations within the laboratory data. Additionally, the scatter plots indicate a substantial correlation with the *R*^2^ values for PET models ranging from 0.75 to 0.90 across all stations, underscoring the need for refining the regression equations to improve model precision. The evaluations indicate that these models provide relatively precise estimations of actual values.

**Table 5:** Performance of ANFIS model

		MSE	RMSE	MAE	NSE	WI	STD
Antalya Centrum	Train	1.84E-05	0.004288	0.3304	0.94572	0.89082	0.41157
	Test	0.0001683	0.0129719	0.66185	0.78845	0.79864	0.51831
Alanya	Train	9.57E-06	0.003093	0.22408	0.94387	0.89536	0.2627
	Test	2.59E-05	0.0050861	0.24855	0.93755	0.88709	0.27413
Finike	Train	1.49E-05	0.003861	0.29287	0.86976	0.8292	0.37441
	Test	4.57E-05	0.0067576	0.33553	0.83433	0.81735	0.39665
Kaş	Train	2.80E-05	0.005295	0.41404	0.86141	0.79532	0.50228
	Test	5.62E-05	0.007499	0.38199	0.87832	0.81034	0.47804
Korkuteli	Train	3.56E-05	0.00597	0.46327	0.8902	0.85584	0.57162
	Test	0.0002212	0.0148735	0.65288	0.71509	0.809	0.85189
Elmalı	Train	4.73E-06	0.002174	0.15524	0.98562	0.94988	0.21075
	Test	1.08E-05	0.0032897	0.15076	0.9856	0.95003	0.20962
Manavgat	Train	1.22E-05	0.00349	0.22247	0.84111	0.84108	0.33335
	Test	2.99E-05	0.0054717	0.23453	0.8348	0.83525	0.33914
Demre	Train	4.58E-05	0.006767	0.50842	0.73996	0.75571	0.65794
	Test	0.0001072	0.0103552	0.5065	0.73794	0.75888	0.65964
Gazipaşa	Train	3.53E-05	0.00594	0.44993	0.78105	0.75	0.56514
	Test	7.92E-05	0.0089011	0.44724	0.79068	0.75402	0.56542

In the third column of Fig. 9, a depiction of the error histogram, MAE, STD, MSE, RMSE, *R*^2^, and ${R}_{adj}^2$values for all datasets are presented. The consistency of *R*^2^ and ${R}_{adj}^2$ values across all stations suggests that the inclusion of additional independent variables does not significantly enhance the model’s explanatory capacity beyond what is already accounted for. When *R*^2^ and ${R}_{adj}^2$ are nearly identical, it indicates that the selected input parameters are aptly chosen and sufficiently explain the desired output. This alignment signifies a well-fitted model where the chosen variables effectively capture the relationships within the dataset without introducing unnecessary complexity or redundancy. The histogram elucidates outliers, representing data points with notably poorer fit compared to most of the dataset. This study observed that while most errors fall within the range of − 2 to 2 across most stations, outliers with errors ranging from − 2.4 to 2.6 exist. These outliers are also evident in the test and training regression plots. Evaluating whether these outliers represent erroneous data points or genuinely distinct observations from the dataset contributes to refining the model training process. When outliers are deemed valid yet atypical, the model may be extrapolating toward these points, underscoring the need for potential retraining to achieve a more accurate fit.

Additionally, if the model performs well on the training set but exhibits significantly poorer performance on the test set, indicative of overfitting, reducing the number of neurons can enhance performance outcomes. Conversely, increasing the number of neurons may enhance training performance metrics in cases where training performance is suboptimal. This underscores the critical role of optimizing weights and biases to promote effective learning during analysis, mitigate challenges such as overfitting and premature convergence, and facilitate streamlined processing without compromising model accuracy.

### Performance of ANFIS-SO model

**Fig. 10:**
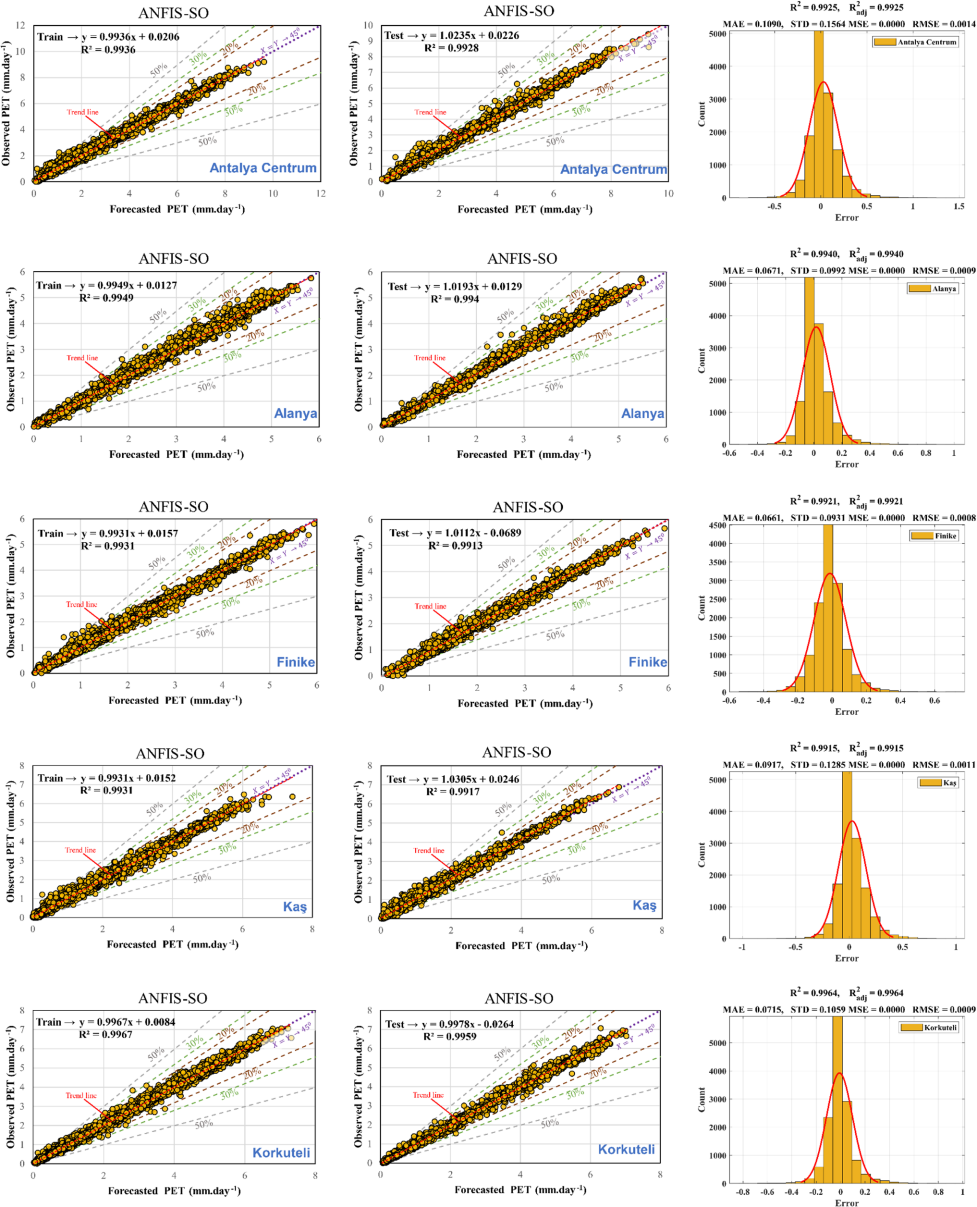

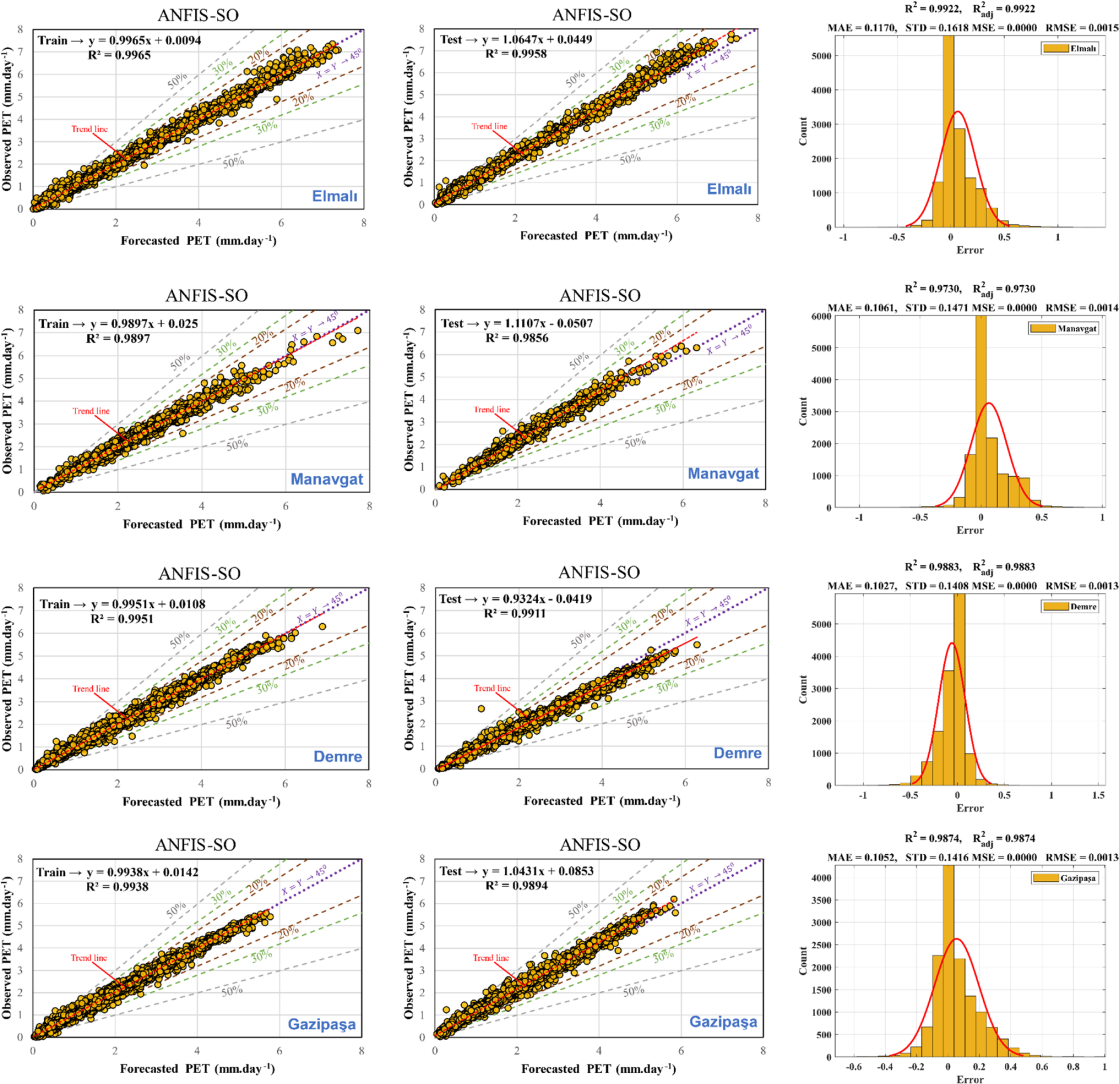
Scatter plot for training and testing phase and histogram errors for all data in ANFIS-SO models

The results of the hybrid method proposed are depicted in Fig. 10 and Table 6. As illustrated in the table, the error values for the training and testing phases significantly decrease compared to the initial state. Furthermore, the non-optimal model’s *R*^2^coefficient, which ranged from approximately 0.75 to 0.91, has improved dramatically to nearly 0.99 (representing a 24% enhancement) in the optimal hybrid model across all stations. Notably, the dispersion of the predicted data during both the test and training phases is below 10%, a substantial improvement from the non-optimal model, where this value stood at approximately 50% for both intervals. Moreover, the standard deviation (STD) values have been notably reduced in comparison to the original solution. The hybrid method demonstrates remarkable enhancements over the non-optimal approach, showcasing superior performance in terms of error reduction, increased accuracy indicated by the *R*^2^ coefficient, decreased data dispersion, and improved consistency, as evidenced by the reduced standard deviation values. These improvements underscore the efficacy and superiority of the hybrid method in addressing the complexities and challenges encountered in the analysis and prediction of the data under consideration.

**Table 6:** Performance of ANFIS-SO model

		MSE	RMSE	MAE	NSE	WI	STD
Antalya Centrum	Train	2.18E-06	0.001476855	0.096636	0.999555	0.96751	0.14382
	Test	8.90E-06	0.00298265	0.13791	0.98891	0.95459	0.16265
Alanya	Train	7.70E-07	0.000877679	0.05825	0.9955	0.97321	0.085469
	Test	1.04E-05	0.003231099	0.16214	0.97456	0.92911	0.1367
Finike	Train	7.98E-07	0.000893068	0.058763	0.99311	0.96615	0.086968
	Test	2.90E-06	0.001703203	0.083254	0.9892	0.95273	0.099592
Kaş	Train	1.38E-06	0.001176393	0.077486	0.99315	0.96653	0.11456
	Test	6.64E-06	0.002576121	0.12481	0.98576	0.94667	0.13625
Korkuteli	Train	1.08E-05	0.003293327	0.067357	0.99669	0.97811	0.10142
	Test	3.38E-06	0.001837362	0.081089	0.99554	0.97357	0.11268
Elmalı	Train	1.16E-06	0.001077311	0.070102	0.99645	0.97712	0.10491
	Test	1.87E-05	0.004324118	0.22659	0.97546	0.92817	0.16709
Manavgat	Train	8.06E-07	0.000897803	0.055282	0.98976	0.96087	0.087429
	Test	1.69E-05	0.004108041	0.22484	0.90127	0.84752	0.14496
Demre	Train	8.57E-07	0.00092584	0.060088	0.9951	0.9722	0.090159
	Test	1.44E-05	0.003789855	0.20223	0.96538	0.90619	0.14465
Gazipaşa	Train	1.01E-06	0.001003245	0.066661	0.99377	0.96807	0.097698
	Test	1.35E-05	0.00367124	0.19513	0.96413	0.90839	0.1436

In the third column of Fig. 10, error histogram graphs are depicted for all datasets, mirroring the structure of the initial model. Notably, the error values have transitioned from a range of − 2.5 to 2.5 to a narrower range of − 0.5 to 0.5, indicating a substantial reduction in errors. This refinement signifies an enhancement in the accuracy and precision of the hybrid model’s predictions. Furthermore, there is a noticeable increase in the *R*^2^ and ${R}_{adj}^2$, underscoring the improved explanatory power and goodness of fit of the model. Moreover, it is noteworthy that the predicted PET values generated by the hybrid models closely align with those calculated by the FAO, indicating high accuracy and reliability in the model's estimations. This alignment with FAO’s established values further validates the hybrid approach’s efficacy and robustness in predicting PET values with greater proximity to ground truth data.

**Fig. 11:**
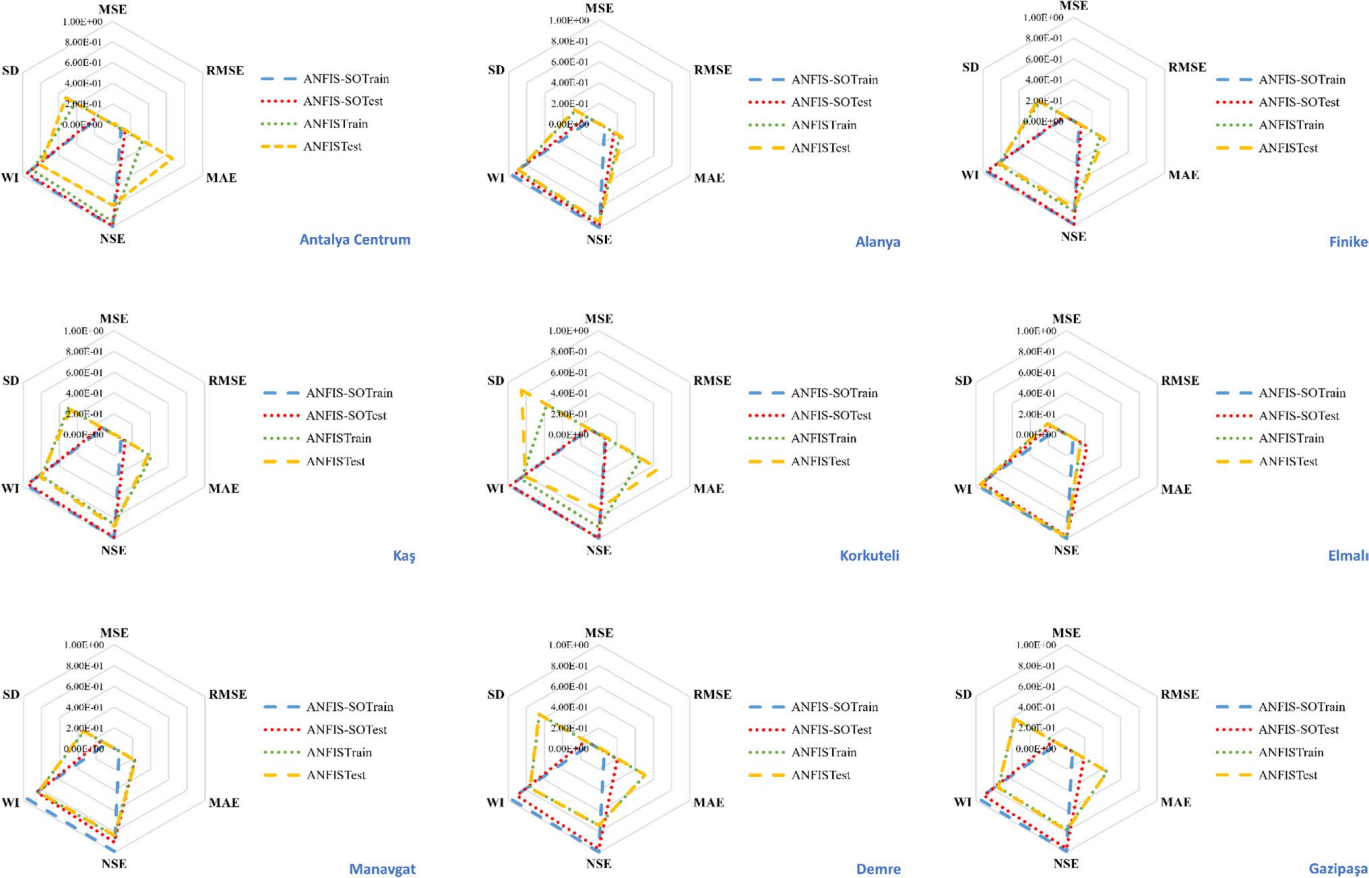
Radar chart to facilitate a comprehensive comparison among the models

Figure 11 depicts the measurement indices in a radar chart format to compare the models comprehensively. The radar chart analysis provides a holistic view of the model’s performance across multiple metrics simultaneously. It offers insights into the strengths and weaknesses of each model, enabling a more nuanced assessment of their overall effectiveness and performance. This visualization aids in highlighting the significant improvements achieved in reducing errors, increasing the coefficient of determination (*R*^2^), and decreasing the standard deviation. The hybrid models have exhibited superior performance compared to their counterparts, showcasing enhanced accuracy, precision, and reliability in predicting outcomes. Utilizing hybrid methodologies has resulted in notable advancements, emphasizing their effectiveness in optimizing model performance and improving predictive capabilities.

### Performance of hybrid model compared with existing analytical methods

**Fig. 12:**
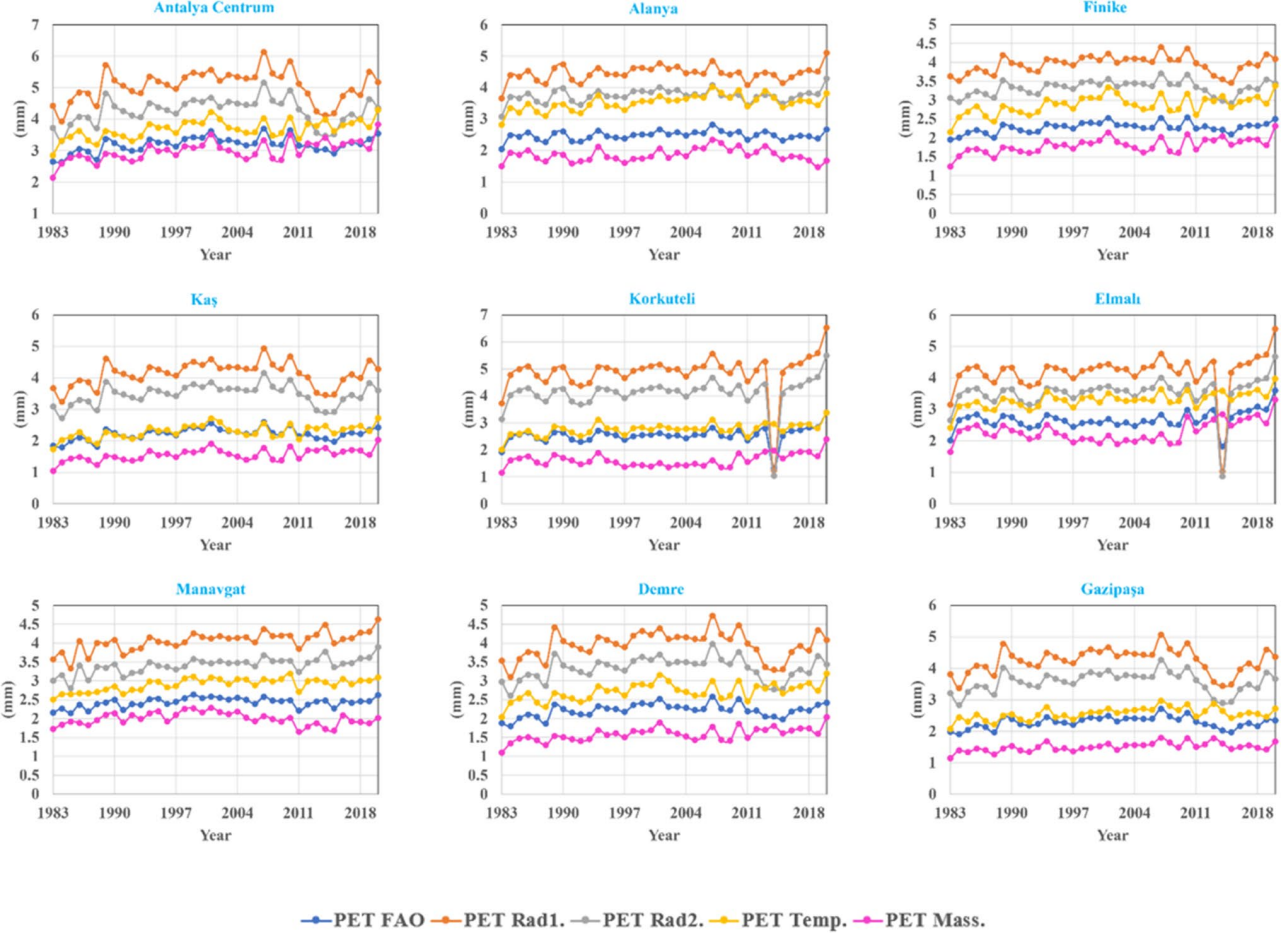
The mean PET values calculated by equations 1 to 3 from 1983 to 2020 across all nine stations (PET_MASS._ = Eq.1, PET_Temp._ = Eq.2, PET_RAD1.a=1.82_ = Eq.3, PET_RAD2.a=2.14_ = Eq.3, and PET_FAO_ = Eq.4)

In this segment, the models illustrated in Table 3 are examined, juxtaposed with the FAO standard equation and the proposed hybrid approach. Figure 12 presents the mean PET values calculated by these equations from 1983 to 2020 across all nine stations. Despite accurately capturing the PET trend through these correlations, a significant margin of error persists compared to the standard benchmark.

**Fig. 13:**
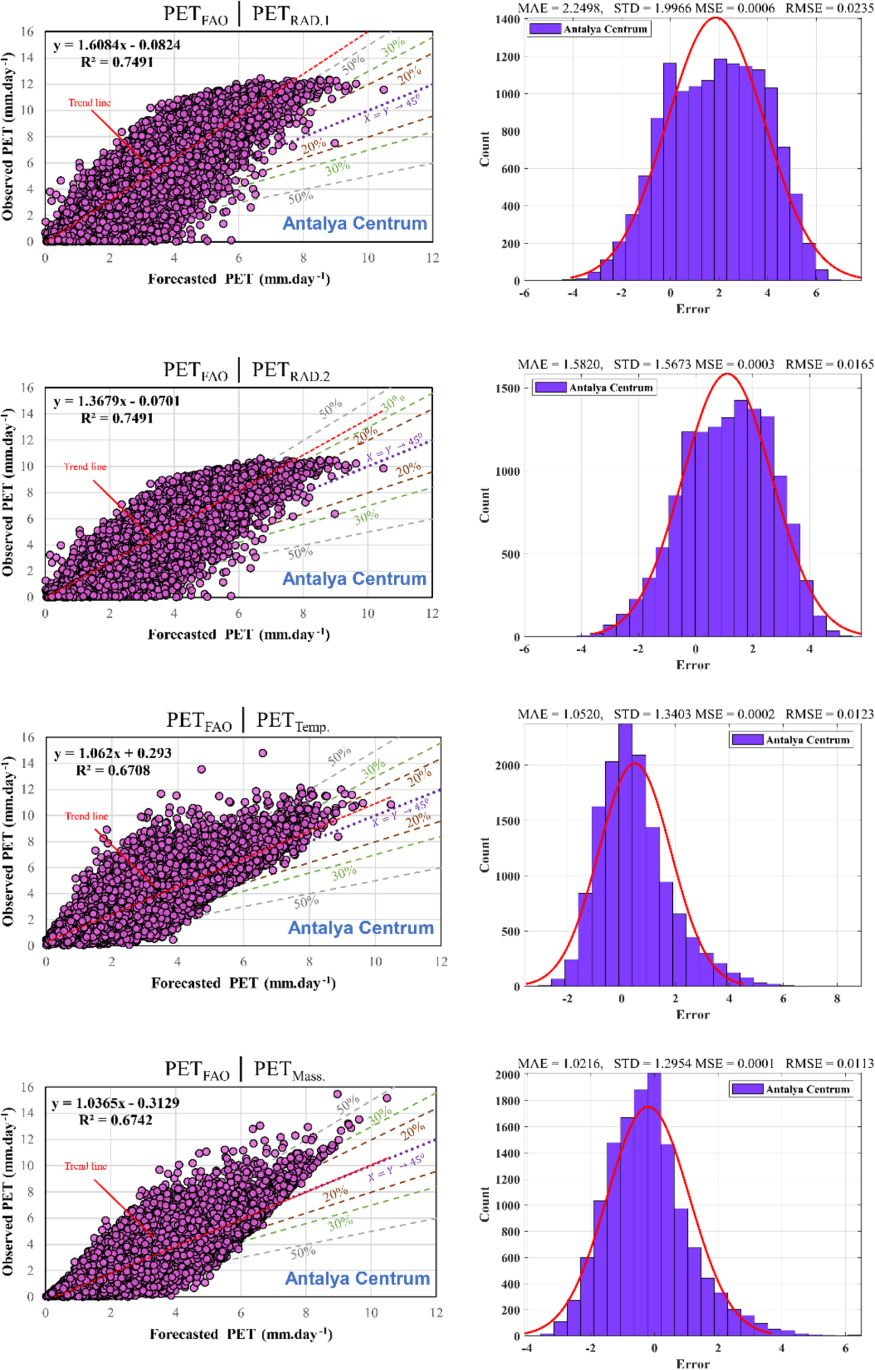
Scatter plot and error distribution for the Antalya Centrum station

Figure 13 illustrates a scatter plot and error distribution for a single station (Antalya Centrum), offering a representative demonstration to provide a more thorough assessment of these discrepancies and model performance. Notably, *R*^2^ reaches a maximum of 0.74. Moreover, the data display notable dispersion, with predicted values deviating over 50% from the ideal fit line and acknowledging that neural network techniques surpass conventional numerical methods due to their advanced learning capabilities in discerning intricate input-output relationships, whether in hybrid or non-hybrid modes. In a scatter plot where the trend line corresponds to the 45° line (*x* = *y*), the R^2^ value should indeed be one. An *R*^2^ value of one indicates that all variability in the dependent variable (Observed data) can be explained by the model's independent variable (forecasted data). If the scatter plot has a trend line corresponding to the 45° line, but the *R*^2^ value is not one (Fig. 13), which suggests that the model does not explain all the variability in the observed data. This means the forecasted data do not account for some degree of variability. For instance, in Fig. 13, PET forecasted by mass transfer-based equation (Eq. 1) has an *R*^2^ value of 0.67, indicating that approximately 67% of the variability in the observed data can be explained by the forecasted data in the model. The remaining 33% of the variability is attributed to other factors not captured by the model. Therefore, even if the trend line aligns with the 45° line, the *R*^2^ value can be less than one if there is still unexplained variability in the data. Due to the proximity and alignment of the trend line with the 45° line, implying a certain level of agreement between observed and forecasted data, an *R*^2^ value less than one in Fig. 13 indicates that there are still unexplained variations in the data that the model does not entirely account for. The overarching objective of this section is to underscore the ongoing necessity for more accurate PET prediction methodologies. Utilizing machine learning approaches and establishing robust input-output relationships may facilitate enhanced predictions and potentially lead to an extrapolation model, enabling assessments over broader time intervals beyond existing dataset limitations.

### Performance of deep learning models

This study’s deep learning method aims to improve the accuracy and precision of potential evapotranspiration predictions compared to the previously employed ANFIS and ANFIS-SO (ANFIS hybrid with Snake Optimizer) approaches. The deep belief networks (DBNs) method captured the temporal patterns and dependencies inherent in the time series data. DBNs have demonstrated strong capabilities in modeling complex non-linear relationships in sequential data. The performance of the DBN deep learning technique was evaluated and compared to the previously reported ANFIS and ANFIS-SO results (based on an unseen data set described in “Data set segmentation” section), providing insights into each approach's relative strengths and suitability for this specific application.

**Fig. 14:**
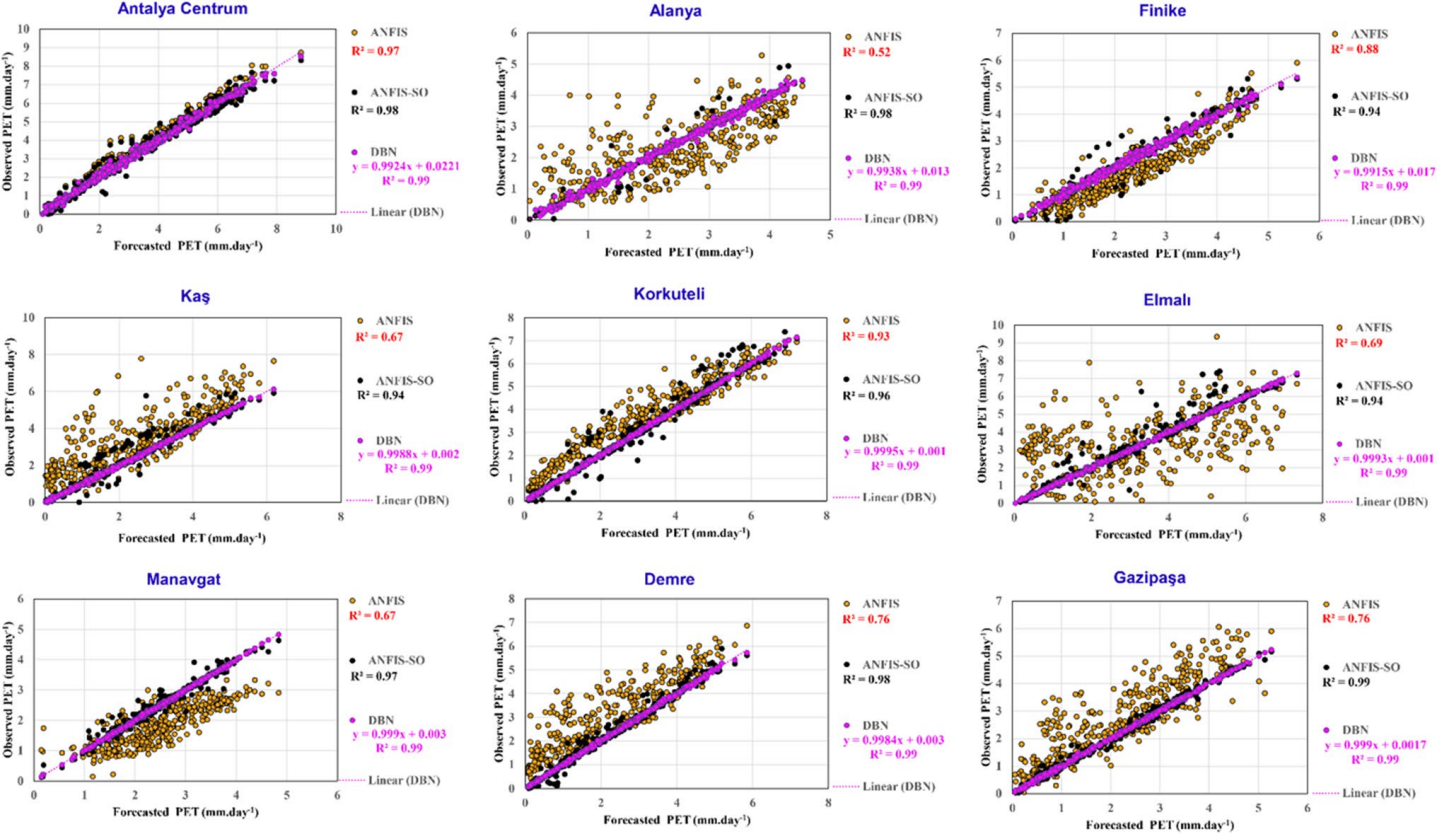
The scatter plot of the ANFIS, ANFIS-SO, and DBN methods across the nine existing stations

The scatter plot in Fig. 14 illustrates the relationship between the observed and predicted data generated by the models across the nine existing stations. As anticipated, the DBN method has demonstrated remarkable performance, successfully providing a robust model for all nine stations with *R*^2^ values approaching unity and remarkably low error metrics. This suggests the DBN model’s exceptional ability to capture the underlying patterns and dynamics inherent within the dataset. In contrast, the ANFIS method has not performed well against this unseen dataset. The ANFIS-SO method, incorporating an SO algorithm, has consistently outperformed the standard ANFIS approach across all the stations.

Starting with Antalya Centrum, the ANFIS method achieved an *R*^2^ value of 0.97, which is already an excellent result. However, the ANFIS-SO method was able to further improve upon this, reaching an *R*^2^ of 0.98. This suggests that the optimization technique employed in the ANFIS-SO method was able to fine-tune the model parameters and enhance the predictive power of the ANFIS algorithm.

The most notable improvement can be seen in the Alanya station, where the ANFIS method had a relatively low *R*^2^ of 0.52, while the ANFIS-SO method achieved an impressive 0.98. This dramatic increase in performance highlights the effectiveness of the optimization algorithm in addressing the limitations of the standard ANFIS model. Similar trends can also be observed in the other stations. The ANFIS-SO method consistently outperformed the ANFIS method, with *R*^2^ values ranging from 0.94 to 0.99 across the remaining stations (Finike, Kaş, Korkuteli, Elmalı, Manavgat, Demre, and Gazipaşa). The ANFIS-SO method’s ability to achieve such high *R*^2^ values, often exceeding 0.90, suggests that the optimization algorithm effectively overcomes the noise and complex relationships between the input variables and the potential evapotranspiration. The optimization process allowed the model to adapt to the unique characteristics of each station, leading to enhanced predictive accuracy.

**Fig. 15:**
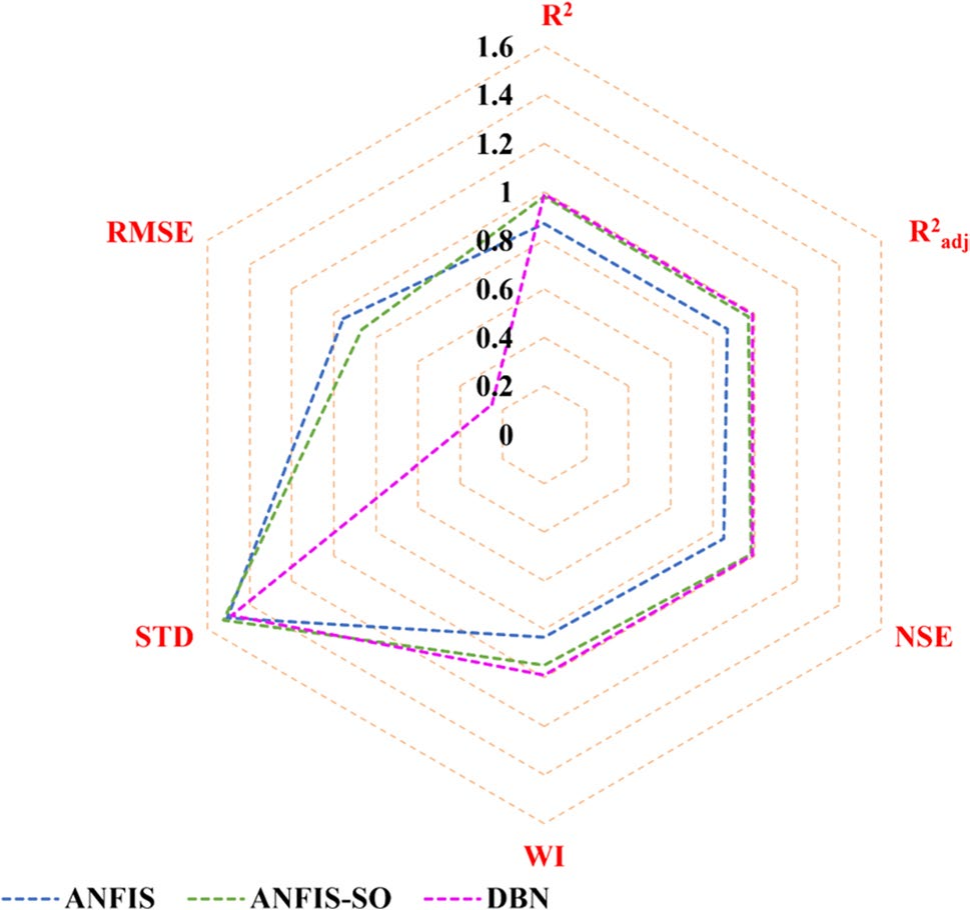
The radar chart across various evaluation metrics, including *R*^2^, *R*^2^_*adj*_, NSE, WI, STD, and RMSE for three proposed models

The radar chart presented in Fig. 15 provides a comprehensive overview of the average performance metrics for DBN, ANFIS, and the optimized ANFIS-SO methods across the nine stations under investigation. The DBN model has outperformed the other two approaches, consistently achieving the highest performance across all the evaluated metrics. With *R*^2^ and ${R}_{adj}^2$ values reaching an impressive 0.99, the DBN method has demonstrated its exceptional ability to capture the underlying patterns and relationships within the data. Furthermore, the NSE and WI scores of 0.99 and 0.99 further validate the DBN's superior predictive accuracy and model reliability. In comparison, the standard ANFIS method, while still exhibiting noteworthy performance, falls short of the DBN’s remarkable achievements. The *R*^2^ and *R*^2^_*adj*_ values of 0.76 and 0.75, respectively, indicate a relatively strong, but not as robust, model fit. Similarly, the NSE and WI scores of 0.65 and 0.63 suggest room for improvement in the ANFIS model’s ability to represent the observed data faithfully. The optimized ANFIS-SO method has managed to bridge the gap, showcasing a substantial enhancement in performance compared to the standard ANFIS. The *R*^2^ and *R*^2^_*adj*_ values of 0.98 and 0.97, respectively, demonstrate the effectiveness of the SO algorithm in fine-tuning the ANFIS model and unlocking its full potential. Moreover, the NSE and WI scores of 0.98 and 0.94 highlight the ANFIS-SO's improved capability to capture the actual underlying dynamics of the system.

The RMSE values further accentuate the comparative performance of the three modeling approaches presented in Fig. 15. The DBN method has achieved an exceptionally low RMSE of 0.248878, indicating its remarkable ability to minimize the discrepancy between the predicted and observed values. This deficient error metric underscores the DBN's prowess in delivering highly accurate and reliable predictions, making it a standout choice for applications demanding precision. In contrast, the standard ANFIS method exhibits a relatively higher RMSE of 0.9559, suggesting a greater degree of uncertainty and deviation from the observed data compared to the DBN. This relatively higher error metric highlights the need for further refinement or optimization of the ANFIS model to enhance its predictive capabilities. In this regard, the ANFIS-SO method has substantially improved RMSE performance, with a value of 0.866867. This significant reduction in the error metric, compared to the standard ANFIS, showcases the effectiveness of the optimization process in enhancing the model’s overall accuracy and robustness.

The RMSE results, when considered alongside the other performance indicators, paint a comprehensive picture of the models’ strengths and weaknesses. The DBN’s exceptionally low RMSE, coupled with its outstanding *R*^2^, *R*^2^_*adj*_ , NSE, and WI scores, solidifies its position as the most reliable and accurate modeling approach among the three. On the other hand, the improved performance of the ANFIS-SO highlights the value of the optimization process in elevating the ANFIS method’s predictive capabilities, bridging the gap with the DBN’s exceptional performance.

**Fig. 16:**
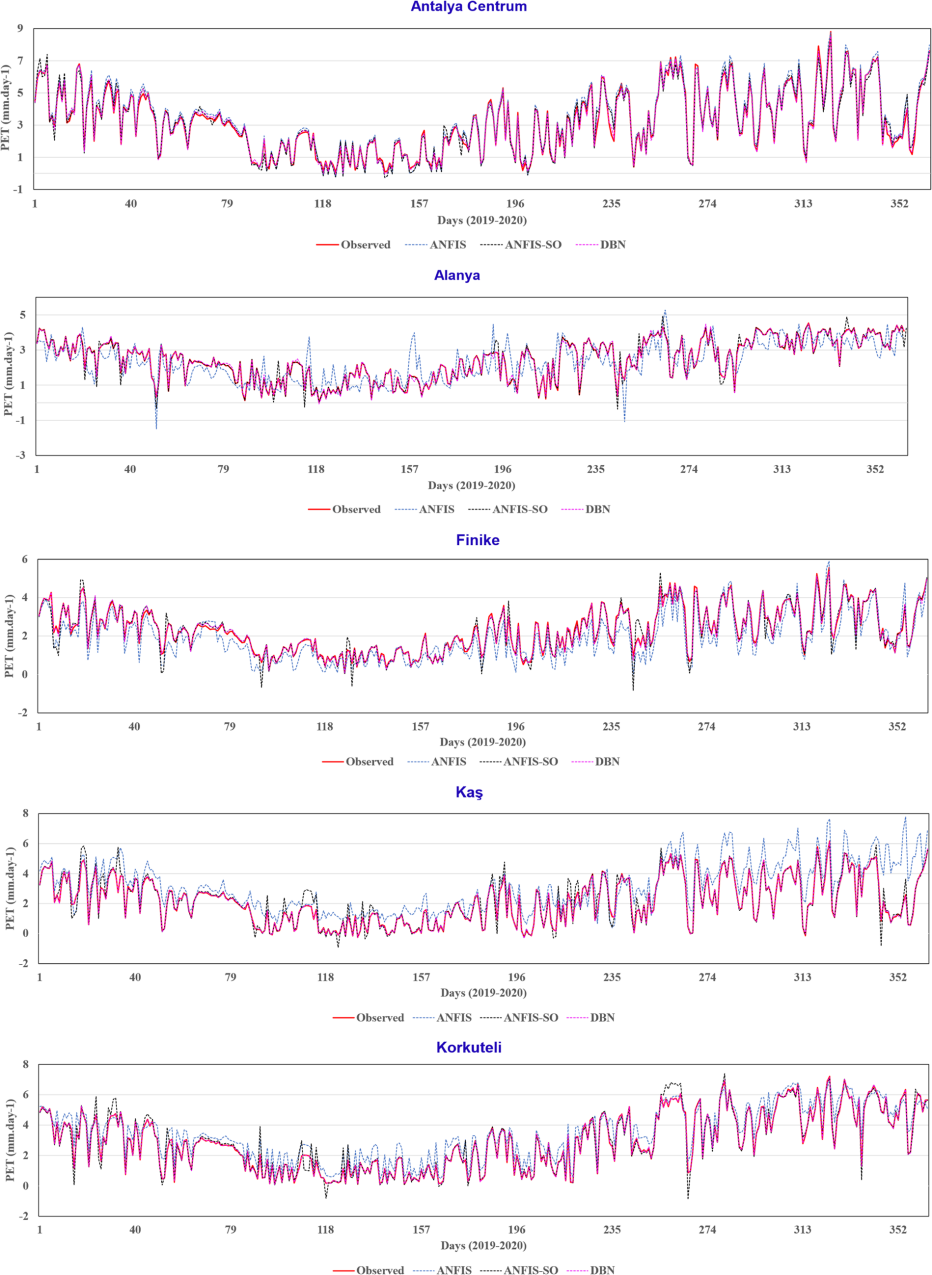

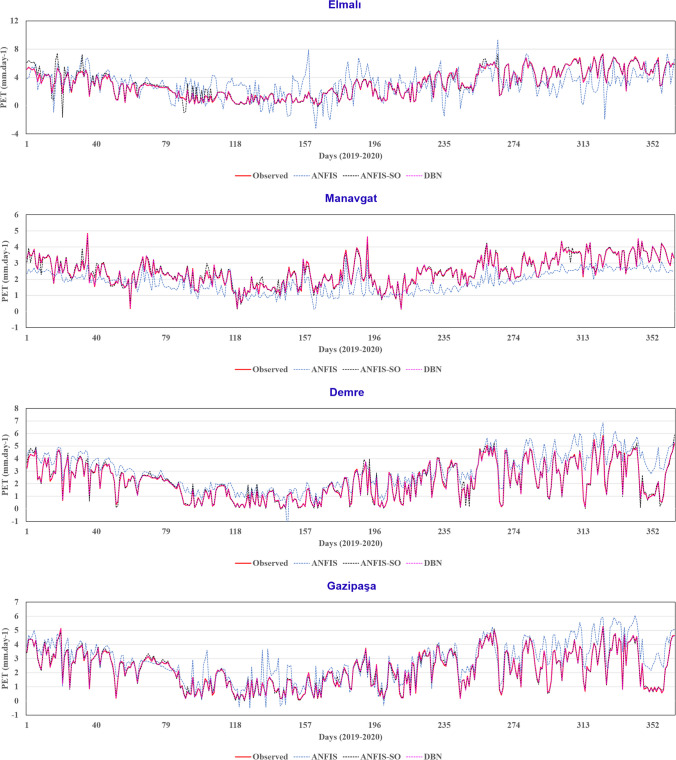
Time history graphs for three models in nine stations

The time history graphs depicted in Fig. 16 provide a comprehensive visualization of the performance characteristics of the three modeling approaches. As anticipated, the DBN model has demonstrated its proficiency in effectively capturing the underlying trends within the observed data. The time history graph showcases the DBN's ability to closely track the fluctuations and accurately predict the peak points, underscoring its robust learning capabilities. Further, the ANFIS method, as evident from the previous scatter plot analysis, has faced challenges in accurately capturing the peak points and valleys within the dataset. The time history graph further accentuates this limitation, revealing the ANFIS model’s struggle to represent the fluctuations inherent in the observed data.

In contrast, the ANFIS-SO method has shown promising improvements in addressing the weaknesses of the standard ANFIS approach. The time history graph indicates that the ANFIS-SO model has minimized the fluctuations and discrepancies observed in the standard ANFIS predictions. However, the graph still suggests the presence of some dispersion and challenges in accurately predicting the peak points, hinting at the need for further optimization. These findings can inform the selection and refinement of modeling strategies, ultimately leading to more reliable and precise predictions, which can have far-reaching implications for decision-making processes across various domains.

**Fig. 17:**
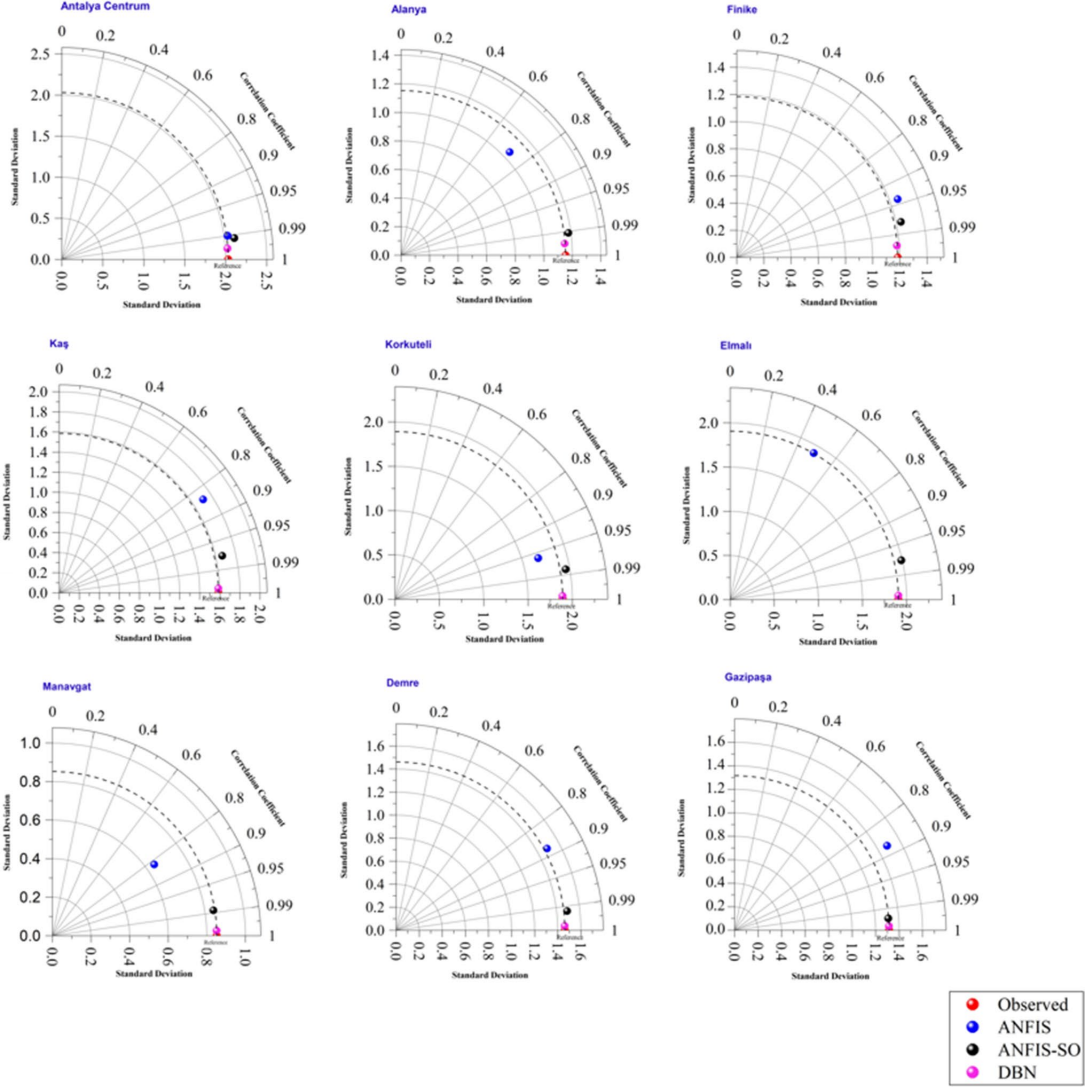
Taylor diagram for three models in nine stations

The Taylor diagram (Fig. 17) depicts the standard deviation and correlation coefficient, providing valuable insights into the models’ ability to capture variability and the degree of association between the observed and predicted values. At the outset, the DBN model emerges as the standout performer, with its data point situated closest to the reference point, indicating an exceptional match between the observed and predicted standard deviations. Moreover, the DBN model exhibits the highest correlation coefficient, approaching unity, signifying its excellent ability to reproduce the observed patterns with high fidelity. The standard ANFIS method displays a more scattered distribution of data points across the Taylor diagram, suggesting a relatively weaker performance regarding both standard deviation and correlation coefficient. This observation aligns with previous findings, where the ANFIS model struggled to accurately capture the fluctuations and peak points within the observed data.

In contrast, the ANFIS-SO method has markedly improved its positioning on the Taylor diagram. The data points for the ANFIS-SO model are notably closer to the reference point and exhibit higher correlation coefficients than the standard ANFIS approach. This underscores the effectiveness of the optimization process in enhancing the model's ability to represent the observed variability better and strengthen the correlation between the predicted and observed values.

**Fig. 18:**
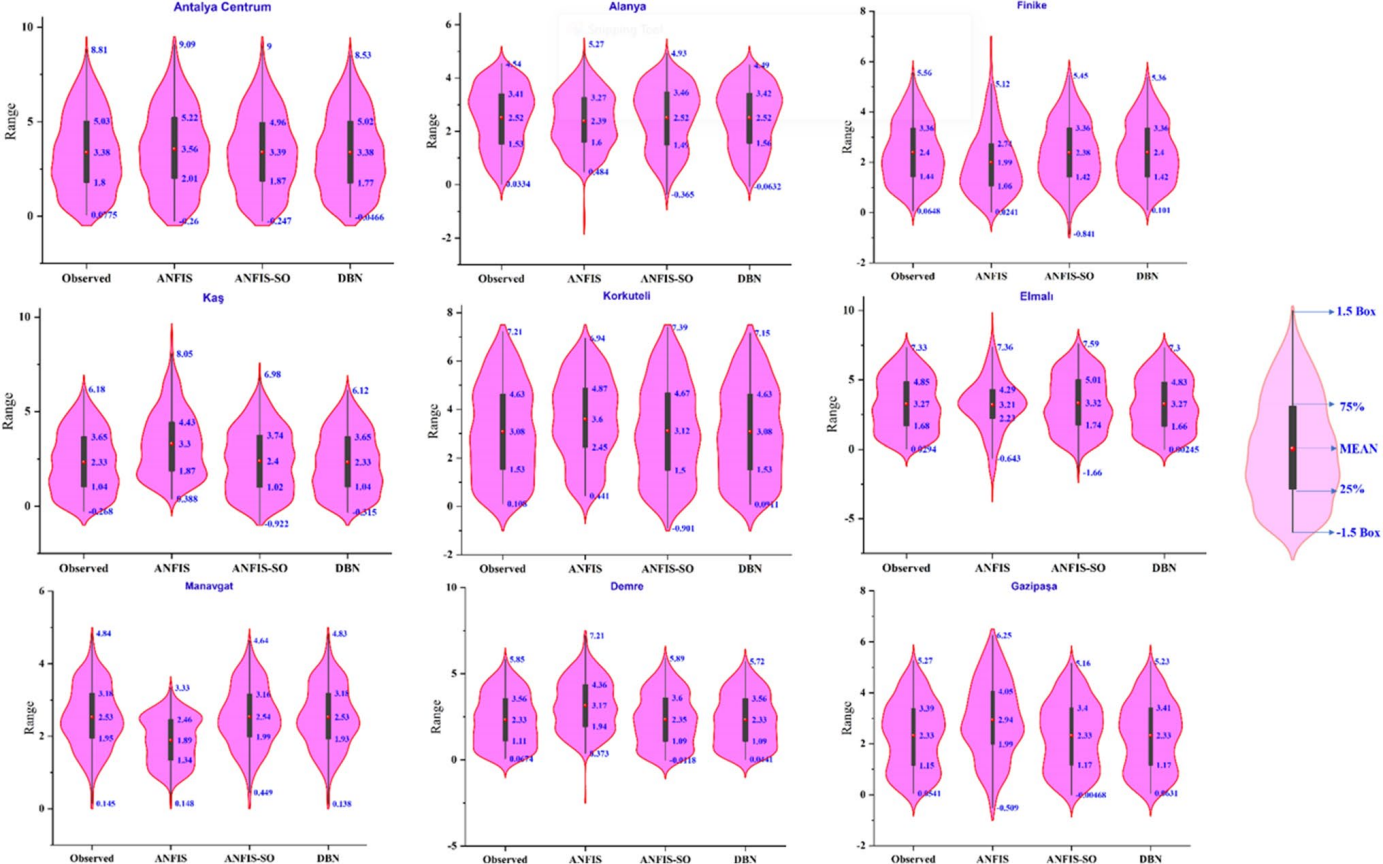
The violin plots with box plots for three models in nine stations

The violin plots with box plots (Fig. 18) presented in the analysis provide a comprehensive visual representation of the statistical distribution and dispersion of the observed and predicted values across the nine stations for the three modeling approaches.

The DBN model’s violin plot exhibits a symmetric shape, indicating a highly concentrated distribution of the predicted values around the mean. This tight clustering of the data points suggests the DBN’s exceptional ability to capture the central tendency and minimize the overall spread of the predictions. In addition, the box plot component further highlights the statistical summary of the DBN model’s performance. The interquartile range (IQR), represented by the box and the whiskers, demonstrates the DBN’s consistency in producing predictions within a tight range with minimal outliers. This observation aligns with the model’s superior *R*^2^, NSE, and WI scores reported earlier, underscoring the DBN’s robustness and reliability.

Further, the ANFIS method’s violin plot exhibits a broader and more asymmetric shape, suggesting more significant variability and dispersion in the predicted values. The box plot for the ANFIS model further reinforces this finding, indicating a higher degree of uncertainty and a more extensive spread of the predictions. The ANFIS-SO model exhibits a more concentrated and symmetric distribution, suggesting that the optimization process has enhanced the model’s ability to reduce variability and better align the predictions with the observed data. The box plot for the ANFIS-SO model further supports previous observations, where the ANFIS-SO method demonstrated superior performance metrics compared to the standard ANFIS approach.

The comparative analysis of the ANFIS, ANFIS-SO, and DBN methods showcases the potential of these approaches in addressing the challenges associated with predicting the target variable. The DBN method’s ability to achieve near-perfect *R*^2^ values and minimal errors suggests its suitability for applications requiring high-precision forecasting. Conversely, the ANFIS-SO method’s strong performance on the unseen dataset demonstrates its robustness and potential for handling diverse scenarios, where flexibility and adaptability are crucial.

The insights from this analysis can have important implications for researchers and practitioners working in predictive modeling. By understanding the strengths and limitations of these techniques, informed decisions can be made regarding the most appropriate modeling approach for a given problem, ultimately leading to enhanced decision-making and more effective resource management.

### Comparison with similar investigations

Artificial intelligence (AI)-based potential evapotranspiration (PET) forecasting has great potential to improve meteorological and hydrological prediction skills. Using more conventional techniques, researchers can use AI to access large datasets and find complex patterns that might not be immediately visible (Vitart et al. 2022). Many methods, including genetic algorithms, artificial neural networks, and remote sensing, have been used to study evapotranspiration forecasting, an essential component of agricultural planning and water cycle knowledge (Valipour 2012). These techniques demonstrate how artificial intelligence (AI) forecasts intricate environmental phenomena. Furthermore, the potential of AI to address environmental concerns across diverse locations is demonstrated by the development of hybrid data-driven machine-learning algorithms for estimating evapotranspiration in varied climates (Valipour et al. 2019).

In several disciplines, including hydrology, environmental science, and agriculture, forecasting potential evapotranspiration (PET) is an essential task. The use of hybrid models that combine the adaptive neuro-fuzzy inference system (ANFIS) with meta-heuristic optimization approaches to improve predicting accuracy has been investigated in many research. The hybrid models of ANFIS and meta-heuristics have demonstrated encouraging outcomes in several applications, such as the prediction of structural load (Ly et al. 2019), rainfall forecasting (Yaseen et al. 2019), and evapotranspiration prediction (Aghelpour et al. 2022). Aghelpour et al. (2022) combined the differential evolution (DE) optimization technique with the ANFIS model to anticipate monthly reference evapotranspiration. The results showed that the hybrid ANFIS-DE model significantly improved ANFIS accuracy by an average of 16%, emphasizing how combining ANFIS with meta-heuristic optimization techniques can increase forecasting precision. The study showed that the hybrid strategy produced good results, highlighting the effectiveness of integrating ANFIS and meta-heuristic optimization.

Furthermore, Alquraish et al. (2021) demonstrated the versatility of combining ANFIS with meta-heuristics across multiple domains by using ANFIS with meta-heuristic algorithms to forecast reservoir inflow forecasting for the King Fahd Dam, Saudi Arabia, which supports the notion that improving predicting results can be achieved by combining ANFIS with meta-heuristic optimization techniques. The hybridization of ANFIS with meta-heuristic optimization algorithms such as genetic algorithm (GA) (Alquraish et al. 2021) and ant colony optimization (ACO) (Aghelpour et al. 2023) has been studied in the context of PET forecasting. As per Mehdizadeh et al. (2021), shuffled frog-leaping algorithm (SFLA) and invasive weed optimization (IWO) have demonstrated their ability to estimate daily reference evapotranspiration accurately. Additionally, research has shown the benefits of hybrid models over conventional methods.

Learnings from research that has successfully applied comparable approaches in different areas may be used in the hybrid methodology of ANFIS and Snake Optimizer algorithm to estimate potential evapotranspiration (PET). Fuzzy logic and neural networks are combined to create ANFIS, which has been successfully hybridized with optimization techniques in various applications. An example of the successful integration of ANFIS with an optimization algorithm for predictive modeling in a particular engineering context is Jithendra (2023), who used ANFIS in conjunction with the Snake Optimizer to model and optimize wire electric discharge machining (WEDM) of Monel 400 alloy.

Pourdaryaei et al. (2019) have also illustrated the efficacy of a hybrid technique that combines the ANFIS with the backtracking search algorithm for short-term power price forecasting. When used for power price forecasting, this hybrid model performed better than conventional optimization strategies, demonstrating the value of combining ANFIS with cutting-edge optimization methods to produce precise forecasts in dynamic systems. Furthermore, a weighted ensemble of ANFIS models for groundwater level prediction based on grey relational analysis and multiple objective genetic algorithms was reported by Roy et al. 2021. This work demonstrated how ANFIS and genetic algorithms may be used to improve hydrological forecasting prediction accuracy, which aligns to optimize PET predictions via hybridization. Maraveas et al. (2022) have emphasized using bio-inspired algorithms, including particle swarm optimization (PSO), to estimate plant evapotranspiration rates. When considering the optimization component of PET forecasting, this reference’s insights on applying optimization algorithms in agricultural engineering may be helpful. Regarding evapotranspiration prediction, these hybrid models outperform traditional ANFIS models.

The current study examined the diverse performance outcomes stemming from applying various optimization methodologies when integrated with the adaptive neuro-fuzzy inference system (ANFIS) predictive models. Specifically, the researchers explored the efficacy of optimization techniques, including genetic algorithm (GA), ant colony optimization (ACO), shuffled frog-leaping algorithm (SFLA), invasive weed optimization (IWO), particle swarm optimization (PSO), and differential evolution (DE), in enhancing the predictive capabilities of the ANFIS framework.

To assess the comparative performance of these optimization-integrated ANFIS models, a suite of evaluation metrics comprising the coefficient of determination (*R*^2^), reliability analysis (RA), and the Nash-Sutcliffe efficiency (NSE). The RA criterion was explicitly defined in Eq. 16, providing a standardized framework for assessing the models’ reliability and consistency.

16 $$\textrm{Reliability}\ \textrm{Analysis}=\textrm{RA}=100\times \left[\frac{1}{r}\times \sum_{i=1}^r{E}_i\right]\kern1em if\kern1em {\displaystyle \begin{array}{cc}\textrm{RAE}\le 0.2& {E}_i=1\\ {}\textrm{Otherwise}& {E}_i=0\end{array}}$$

Where *r* represents the number of rows and the variable *E*_*i*_ is calculated based on the relative average error (RAE).

**Fig. 19:**
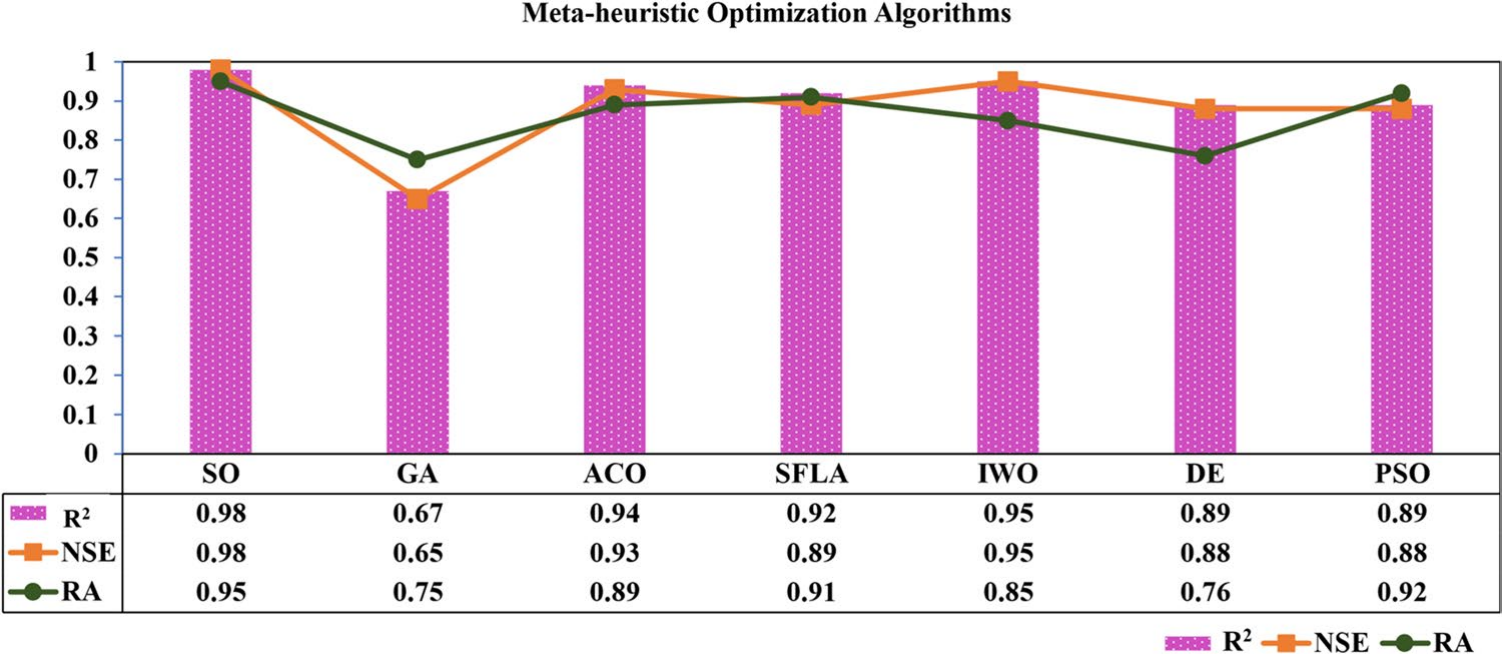
R^2^, NSE, and RA terms to compare the performance of the meta-heuristic optimization algorithms

The results of this comparative analysis, as depicted in Fig. 19, represent the average performance metrics obtained from 50 distinct simulation runs (for nine stations). Notably, the findings in this figure pertain to the unseen dataset, offering valuable insights into the models’ generalization abilities and capacity to forecast previously unencountered scenarios accurately.

According to Fig. 19, the Snake Optimizer (SO) emerges as the standout performer, achieving the highest scores across all three evaluation criteria. The SO-enhanced ANFIS model attained an impressive *R*^2^ of 0.98 and an NSE of 0.98, indicating its exceptional capacity to capture the underlying patterns and dynamics within the dataset. Furthermore, the RA score of 0.95 underscores the model's reliability and consistency in its predictions.

In contrast, the genetic algorithm (GA)-optimized ANFIS model exhibited the weakest performance, with an *R*^2^ of 0.67, NSE of 0.65, and RA of 0.75. This suggests that the GA optimization approach was less effective in enhancing the ANFIS model's predictive capabilities compared to the other techniques examined.

The remaining optimization algorithms, namely ACO, SFLA, IWO, DE, and PSO, demonstrated intermediate levels of performance with *R*^2^ and NSE values ranging from 0.88 to 0.95. The ACO and IWO methods, in particular, displayed relatively more robust performance, with *R*^2^ and NSE scores exceeding 0.90, indicating their potential to improve the ANFIS model’s predictive accuracy.

The Snake Optimizer’s superior performance highlights its suitability for enhancing the ANFIS framework, particularly in applications where accurate forecasting and reliable predictions are paramount. Conversely, the genetic algorithm’s underperformance suggests further investigation and refinement of this optimization approach when applied to ANFIS-based models. The comparative analysis presented in this study can guide the selection of the most appropriate optimization strategy based on the specific requirements and constraints of the problem.

## Conclusions

In this study, the DBN, ANFIS, and ANFIS-SO models were employed to forecast potential evapotranspiration (PET), utilizing various evaluation metrics to assess the accuracy of the predictive models. An array of evaluation metrics, including the coefficient of determination (*R*^2^), adjusted coefficient of determination (${R}_{adj}^2$), mean square error (MSE), root mean square error (RMSE), mean absolute error (MAE), Nash-Sutcliffe efficiency (NSE), Wilmot Index (WI), and standard deviation (STD), were harnessed to scrutinize the predictive capabilities of the models. The findings of this research endeavor can be categorized into three distinct components:

1. Performance evaluation of standard ANFIS and optimized ANFIS models during training and testing:
  The ANFIS-SO model demonstrated superior performance with maximum metric values of MSE = 1.87E-05, RMSE = 4.32E-03, MAE = 2.27E-01, NSE = 0.99, WI = 0.97, and STD = 1.67E-01. In some stations, the scattering of data compared to the ideal fit line was 10% or less, indicating a substantial enhancement in predictive accuracy. In contrast, the ANFIS model exhibited metric values with MSE = 2.21E-04, RMSE = 1.49E-02, MAE = 6.62E-01, NSE = 0.98, WI = 0.95, and STD = 8.52E-01. The scattering of data compared to the ideal fit line was above 40% for all stations, suggesting a lower level of accuracy compared to the optimized ANFIS-SO model. The most notable improvement was observed in the *R*^2^ coefficient increased significantly from approximately 0.75 to 0.91 in the non-optimal model to nearly 0.99 in the optimal hybrid model across all stations, representing a remarkable 24% enhancement in predictive capability.

2. Comparative analysis of optimized ANFIS and deep learning approaches on unseen data:

The analysis of the performance of the DBN model on the unseen dataset reveals its exceptional capabilities. The DBN consistently achieved near-perfect *R*^2^ and ${R}_{adj}^2$ values of 0.99 across the nine stations examined, demonstrating its remarkable ability to capture the intricate complexities and patterns within the dataset, enabling exact predictions even on previously unencountered data. The DBN also maintained superior performance regarding the NSE and WI, reaching 0.99, indicating the model’s exceptional reliability and alignment with the observed data.

In contrast, the standard ANFIS method, while still exhibiting noteworthy performance, displayed relatively weaker results when confronted with the unseen dataset. *R*^2^ and ${R}_{adj}^2$ values ranged from 0.52 to 0.88 across the stations, suggesting that the model struggled to capture the nuances and variations within the data fully.

Notably, the ANFIS-SO method improved its predictive capabilities on the unseen data. The *R*^2^ and ${R}_{adj}^2$ values for the ANFIS-SO model ranged from 0.94 to 0.99, showcasing the effectiveness of the optimization process in enhancing the model's ability to generalize and adapt to previously unencountered conditions.

The comparative analysis of the RMSE values further reinforces these findings. The DBN model achieved an exceptionally low RMSE of 0.248, demonstrating its remarkable capacity to minimize the discrepancy between predicted and observed values. The ANFIS-SO method also significantly improved, with an RMSE of 0.866, outperforming the standard ANFIS method’s RMSE of 0.955.

3. Enhancing standard ANFIS performance through algorithms from previous studies:

The comparative analysis of the optimization algorithms integrated with the ANFIS model revealed notable disparities in their performance. The Snake Optimizer (SO) emerged as the top performer, achieving exceptional *R*^2^, NSE, and RA of 0.98, 0.98, and 0.95, respectively. In contrast, the genetic algorithm (GA) exhibited the weakest results, with comparatively lower metric values of 0.67, 0.65, and 0.75. The remaining optimization techniques, including ant colony optimization (ACO), shuffled frog-leaping algorithm (SFLA), invasive weed optimization (IWO), differential evolution (DE), and particle swarm optimization (PSO), demonstrated intermediate levels of performance, with *R*^2^ and NSE ranging from 0.88 to 0.95, indicating their varying degrees of effectiveness in enhancing the ANFIS model’s predictive capabilities.

Adding an optimization algorithm to the ANFIS structure has enhanced the results by improving key metrics such as MSE, RMSE, MAE, NSE, WI, and STD. The optimization method proved essential in refining the model’s accuracy and predictive performance, leading to more reliable forecasts of potential evapotranspiration. The advantages of employing an optimization algorithm include increased precision in predictions, reduced errors (as reflected in lower MSE, RMSE, and MAE values), enhanced model efficiency (as indicated by higher NSE and WI values), and improved consistency (as shown by lower STD values). These benefits highlight the significance of optimization techniques in enhancing the performance of predictive models and underscore their importance in research and practical applications.

This study’s findings emphasize the critical role of optimization algorithms in improving the accuracy and reliability of predictive models for forecasting potential evapotranspiration. The substantial enhancements observed in key evaluation metrics underscore the effectiveness of incorporating optimization methods into hybrid models like ANFIS-SO, paving the way for more precise and dependable environmental and hydrological study predictions. The results show that ANFIS-SO performs comparably to existing PET forecasting models and can give practical and accurate solutions to engineering optimization challenges. These models may be utilized as an effective supplementary tool for generating precise evapotranspiration while saving money and effort. Another avenue of research is to apply the ANFIS-SO model to various optimization problems and investigate its benefits and drawbacks. ANFIS-SO will be used in the future to handle a variety of challenges, including water quality analysis, water flow rate, sediment temperature, sediment discharge, and other practical engineering issues.

## Data Availability

Data are available from the authors.
